# Glutamate: Molecular Mechanisms and Signaling Pathway in Alzheimer’s Disease, a Potential Therapeutic Target

**DOI:** 10.3390/molecules29235744

**Published:** 2024-12-05

**Authors:** Nidhi Puranik, Minseok Song

**Affiliations:** Department of Life Sciences, Yeungnam University, Gyeongsan 38541, Republic of Korea

**Keywords:** glutamate, Alzheimer’s disease, neurotransmitter, NMDAR antagonist, acetylcholinesterase inhibitors

## Abstract

Gamma-glutamate is an important excitatory neurotransmitter in the central nervous system (CNS), which plays an important role in transmitting synapses, plasticity, and other brain activities. Nevertheless, alterations in the glutamatergic signaling pathway are now accepted as a central element in Alzheimer’s disease (AD) pathophysiology. One of the most prevalent types of dementia in older adults is AD, a progressive neurodegenerative illness brought on by a persistent decline in cognitive function. Since AD has been shown to be multifactorial, a variety of pharmaceutical targets may be used to treat the condition. N-methyl-D-aspartic acid receptor (NMDAR) antagonists and acetylcholinesterase inhibitors (AChEIs) are two drug classes that the Food and Drug Administration has authorized for the treatment of AD. The AChEIs approved to treat AD are galantamine, donepezil, and rivastigmine. However, memantine is the only non-competitive NMDAR antagonist that has been authorized for the treatment of AD. This review aims to outline the involvement of glutamate (GLU) at the molecular level and the signaling pathways that are associated with AD to demonstrate the drug target therapeutic potential of glutamate and its receptor. We will also consider the opinion of the leading authorities working in this area, the drawback of the existing therapeutic strategies, and the direction for the further investigation.

## 1. Introduction

**Figure 1:**
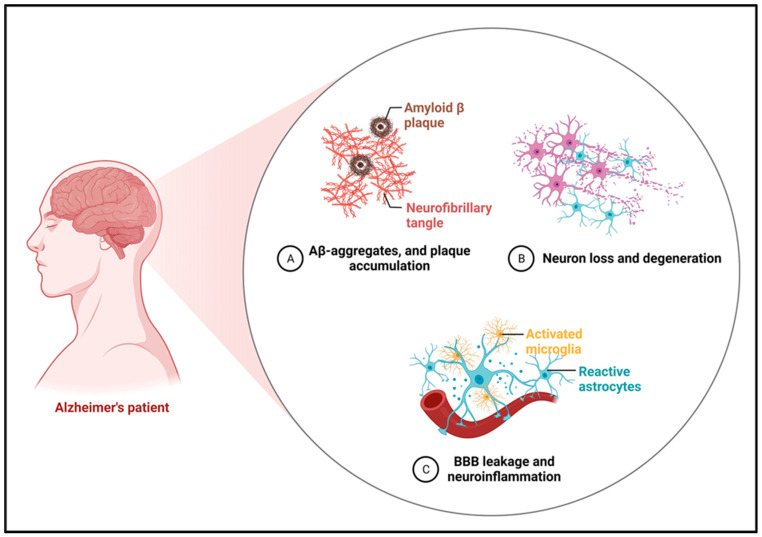
The figure depicts the main pathogenic features of Alzheimer’s disease, including Amyloid-beta plaques, neurofibrillary tangles, neuronal damage, decline, blood–brain barrier leakage, and neuroinflammation (created by Biorendor.com).

Alzheimer’s disease (AD) is a chronic progressive condition affecting memory and cognition that most frequently accounts for dementia in the global population [1]. It is associated with spatial and chronic, slowly evolving memory impairment and profound neuronal depletion leading to the state of dementia and dramatically reduced life quality, as well as incapability in performing the simplest tasks. In today’s world, significant attempts have been made to understand the causes of AD; however, the exact cause of the ailment remains unknown. The disease is associated with a variety of pathological processes [2]. AD is also characterized by the deposition of extracellular amyloid-beta (Aβ) plaques, intracellular neurofibrillary tangles (NFT) formed from hyperphosphorylated tau and neuroinflammation that leads to neuron damage [3], as shown in Figure 1. Microglia and astrocytes are key players in the brain’s neuroinflammatory response, working together at various stages of brain pathology. Microglia, often described as sentinels of the brain, actively monitor their environment. When they detect signs of damage, they become activated through signaling molecules, which, in turn, stimulate reactive astrocytes. Astrocytes, positioned strategically to mobilize the immune system, amplify neuroinflammation through their functions. In AD, the abnormal behavior of these glial cells contributes to disruptions in neuronal calcium (Ca^2+^) balance and hinders the clearance of protein aggregates, such as Aβ plaques. These disruptions impair neuronal function and survival, leading to cognitive decline. The altered genetic profiles, cellular responses, and molecular interactions of glial cells exacerbate immune dysregulation and dysfunctional cross-talk, worsening AD pathology [4,5,6].

**Figure 2:**
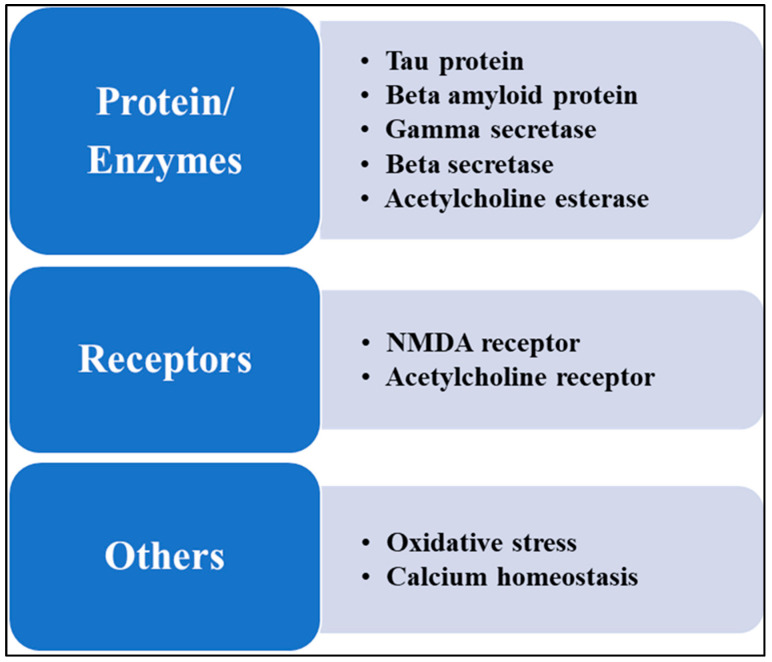
The picture focuses on Alzheimer’s disease treatment objectives. Tau and beta-amyloid proteins, which are essential for pathogenic aggregation, gamma-secretase and beta-secretase, which are enzymes involved in the synthesis of amyloid, and acetylcholine esterase, a target for improving cholinergic function. The N-methyl-D-aspartate receptor (NMDA) and cholinergic receptors, which are intended to alter neuronal signaling and lessen excitotoxicity, and oxidative stress and calcium homeostasis, are major targets for AD therapeutics.

These pathological changes relate to synaptic disorders, neuronal loss, and deleterious cognitive dysfunctions. Pathogenic proteins and enzymes involved in the synthesis and regulation of these proteins are major therapeutic targets of AD; however, lipid receptors, neurotransmitter receptors, oxidative stress, and dysregulated calcium homeostasis are also targeted for AD treatment and management [7,8], as shown in Figure 2.

**Figure 3:**
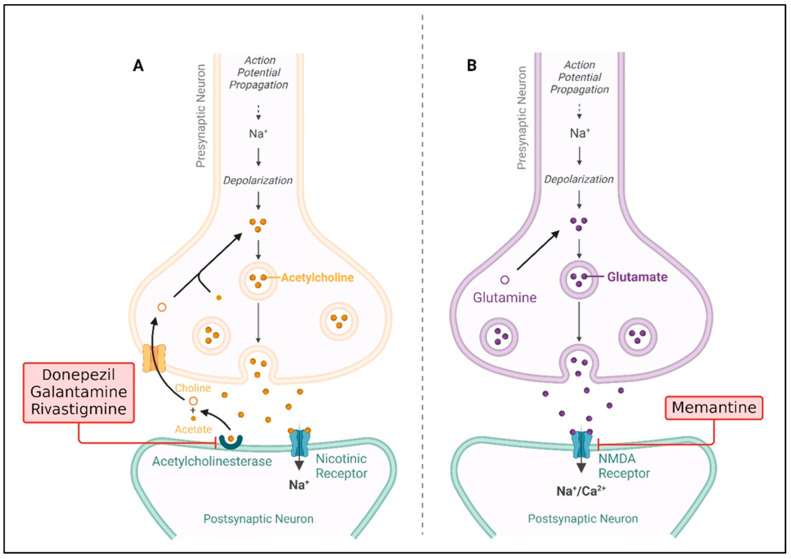
FDA-approved drug treatment strategies for Alzheimer’s disease. (**A**) The drugs donepezil, galantamine, and rivastigmine inhibit acetylcholinesterase, preventing the breakdown of acetylcholine and thus enhancing cholinergic signaling. (**B**) The drug memantine blocks NMDA receptors, preventing excessive calcium influx that could lead to neuron damage (created by Biorendor.com).

Nevertheless, in recent years, the importance of the neurotransmitter systems, particularly the glutaminergic system, in the pathophysiology of AD has been confirmed [9,10,11]. Outside of the peripheral nervous system, glutamate (GLU) is the excitatory neurotransmitter, and plays crucial roles in learning, memory processes, and synaptic plasticity in the central nervous system (CNS) [12,13]. The effects are mediated through several receptors, including ionotropic glutamate receptors (iGLURs)-N-methyl-D-aspartate receptor (NMDA), α-amino-3-hydroxy-5-methyl-4-isoxazolepropionic acid (AMPA) receptors and metabotropic glutamate receptors (mGLURs). GLU signaling is an important constituent in the normal functioning of the brain, and its over- or under-stimulation can lead to devastating effects [13,14]. However, the malfunctioning of this system will give rise to excitotoxicity, a condition whereby increased glutamate receptor (GLUR) activity is toxic to neurons. This excitotoxic damage has been also associated with various diseases of a neurodegenerative nature, such as AD [15,16,17,18,19,20]. Data from recent years indicate that modifications in the GLU concentration and functionality might be at the heart of disease development in AD [21]. Several studies have pointed out that an increased concentration of GLU outside the cells, abnormal stimulation of the receptors, and failure to remove GLU from the environment are linked with neuronal damage and death in brains affected by AD [22,23]. Also, the combination of Aβ peptides with the glutamatergic receptors makes the situation worse by worsening the situation in synapses, and thus cognition. For example, Aβ was identified to increase NMDA receptor function, resulting in an influx of calcium and neuronal toxicity, while the tau pathology negatively affects mGluR signaling, thereby worsening synaptic plasticity [24,25,26]. Based on these findings, the approach that attacks the overstimulation of the glutamatergic system became the most effective treatment for AD. Only five medications have received FDA approval to treat AD thus far. Four of these five medications—donepezil, galantamine, rivastigmine, and tacrine—are AChE inhibitors (AChEIs), and memantine is the only NMDAR antagonist (as shown in Figure 3) that has been approved [27,28,29,30,31,32]. Memantine, which is an NMDA receptor antagonist, seeks to control excitotoxicity while giving only symptomatic relief [33]. However, these are not very effective and do not slow down the disease development. This highlights the need for simple molecular studies providing insight into GLU dysfunction in AD in the design of stringer therapeutic approaches. The present review aims to discuss the evidence that points to GLU as involved in the molecular mechanism of AD [34]. We adopted a narrative review approach, focusing on the relevant literature by searching the keywords glutamate, Alzheimer’s disease, and GLU as therapeutic targets on platforms such as Google Scholar and PubMed. We aimed to identify key studies that discuss the role of GLU in AD and its potential as a therapeutic target. Articles were selected based on their relevance, novelty, and contributions to understanding this topic. In the further sections, we will consider different subtypes of GLURs and transporters, and signaling pathways involved in the pathogenesis of AD. Additionally, we will analyze available and experimental treatment approaches that involve GLU signaling and their advantages and disadvantages. By dissecting the complex interplay between GLU dysregulation and AD pathology, we aim to shed light on novel therapeutic targets and future research directions that may pave the way for more effective treatments for this devastating disease.

## 2. Glutamate Synthesis and Metabolism

The main precursor of GLUin the brain is glutamine (GLN), a key component of the glutamine–glutamate cycle, and this reaction is catalyzed by the enzyme glutaminase (GLS), which is largely present in presynaptic neurons. Synaptic GLU participates in a highly active recycling process between presynaptic glutamatergic neurons and astrocytes, known as the glutamate–glutamine cycle [35,36,37,38]. Depending on their localization and function, the glutaminase enzymes are divided into two primary isoenzymes, namely, kidney-type glutaminase (GLS1) and liver-type glutaminase (GLS2). GLS1 is particularly dominant in neurons and is highly involved in neurotransmission [39].

Most of the GLU activity is localized in the mitochondria of neurons; therefore, the conversion of GLN to GLU occurs in the mitochondria [40]. This is important due to the requirement for a constant supply of this excitatory neurotransmitter for the refilling of synaptic vesicles. The reaction involves the removal of an amine group from GLN, resulting in the formation of GLU and free ammonia (NH_3_) [41].

Nonetheless, there exists a minor pathway for the synthesis of GLU in neurons through the transamination of α-ketoglutarate, which is a part of the tricarboxylic acid cycle or the Krebs’ cycle, which is involved in the energy metabolism of the cell [42]. Transamination involves the transfer of an amino group to α-ketoglutarate, forming GLU and a mish-mash keto acid. This reaction is catalyzed by enzymes such as alanine aminotransferase (ALT) and aspartate aminotransferase (AST) [43,44].

**Figure 4:**
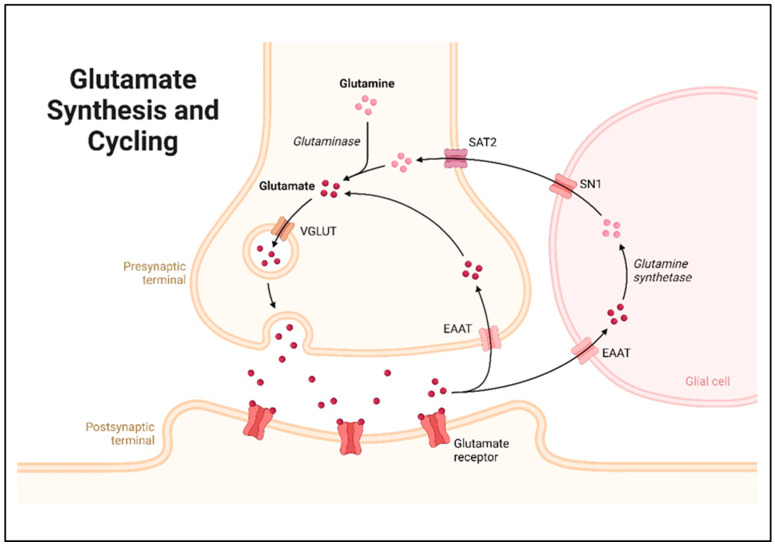
Diagrammatic representation of glutamate synthesis and cycling in neurons. This cycle helps maintain glutamate levels in the synapse, supports neurotransmission, and prevents excitotoxicity. The roles of EAAT, SN1, and SAT2 in the cycling process are critical for transferring glutamate and glutamine between neurons and glial cells, highlighting the cooperative nature of neurons and glial cells in regulating neurotransmitter levels (created by Biorendor.com).

After synthesis, GLU resides in synaptic vesicles in the presynaptic neuron, wherein it waits for a signal to be released and commence its action. GLU packaging into these vesicles involves vesicular glutamate transporters (VGLUTs), which include VGLUT 1, VGLUT 2 and VGLUT 3 [45]. The transporters take up Glu into synaptic vesicles thermodynamically against its concentration gradient through the utilization of a vesicular ATPase. When an action potential reaches the presynaptic terminal, the voltage-sensitive calcium channels of the membrane open. These ions allow synaptic vesicles to merge with the presynaptic membrane through a process called exocytosis, and this process releases the neurotransmitter called GLUinto the synaptic cleft [46]. After initiating the signaling cascade, glutamate molecules are reabsorbed by pre-synaptic neurons and glia cells by excitatory amino acid transporters (EAATs) and take part in a further cycle [47]. Under normal circumstances, astrocytes strictly regulate the glutamate equilibrium in the neuropil. The primary pathway for extracellular GLU elimination is the expression of EAAT proteins by astrocyte processes around glutamatergic synapses. The enzyme glutamine synthetase, which is predominantly expressed in astrocytes, converts most GLU to GLN. An electroneutral, sodium-dependent transporter called SN1 then releases glutamine from astrocytes into the extracellular space, where it is subsequently absorbed by system A transporters (SAT2) and transported into neurons. GLU and GABA, two active neurotransmitters that are packed inside vesicles and released again during synaptic transmission, are synthesized in neurons using glutamine as a precursor [48,49]. A basic schematic representation of GLU synthesis and cycling is shown in Figure 4.

## 3. Glutamate Signaling and Receptor Activation

**Figure 5:**
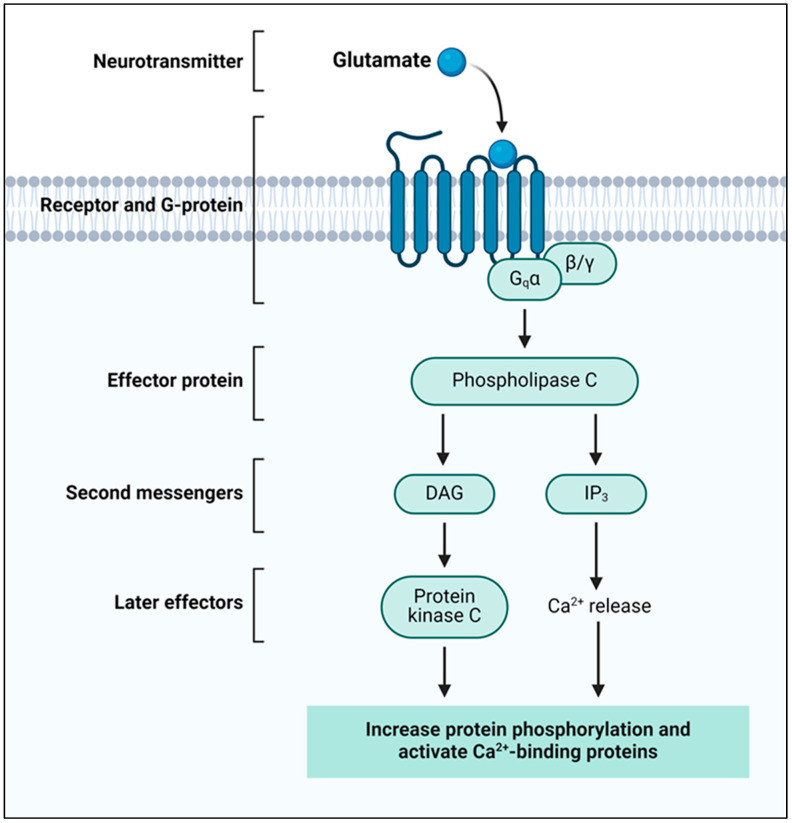
Diagram depicting the glutamate-mediated G-protein coupled receptor (GPCR) signaling pathway. The effector protein phospholipase C (PLC) is stimulated when the neurotransmitter glutamate binds to its receptor and activates a G-protein (Gqα). Two second messengers, inositol triphosphate (IP_3_) and diacylglycerol (DAG), are produced by PLC. IP_3_ causes the release of Ca^2+^ ions from intracellular storage, whereas DAG activates protein kinase C (PKC). When combined, these mechanisms result in elevated protein phosphorylation and Ca^2+^-binding protein activation, which promote downstream cellular reactions involved in a number of physiological functions (created by Biorendor.com).

Glutamate is the primary positively charged neurotransmitter in the CNS and plays an important role in synaptic transmission. After being released into the synaptic cleft, GLU attaches to specific receptors on the membrane of the postsynaptic neuron, initiating specific intracellular changes [13,50]. The classification of glutamate receptors is as follows: ionotropic glutamate receptors (iGLURs) and metabotropic glutamate receptors (mGLURs) are the two major classes of glutamate receptors [51,52,53,54]. iGLURs are ligand-gated ion channels that mediate fast synaptic transmission. When GLU binds to these receptors, they undergo a conformational change, allowing ions to flow across the neuronal membrane [55]; a basic representation of GLU signaling is shown in Figure 5. The main subtypes of iGLURs include NMDA and AMPA receptors.

NMDA receptors consist of a family of transmembrane proteins comprised of NR1 and NR2 transcription products, and play an essential role in synaptic transmission and plasticity in the CNS [56,57]. These receptors have a specific activation process that involves the need for GLU and a co-agonist like glycine or D-serine, making them more sensitive to GLU [58]. This requirement means that the NMDA receptors are activated only when there is intense synaptic activation, hence avoiding over-excitation. Primarily, the NMDA receptors are of the voltage-dependent type, as magnesium ions (Mg^2+^) inhibit its action during the membrane resting potential [59]. This inhibition is released upon depolarization, such that the ion passes through the receptor channel. When opened, NMDA receptors permit the entrance of sodium (Na^+^) and calcium (Ca^2+^) ions, as well as the release of potassium (K^+^) ions. The operation of Ca^2+^ is even more important; it is Ca^2+^ that can often work as a second messenger crucial for the intracellular signaling that is required for synaptic plasticity. This includes the ability to stimulate long-term potentiating, which enhances the synaptic connections between the neurons, and long-term depression, which reduces them [60]. The above is crucial in learning and memory; these processes help the brain to modulate depending on experience. The fine-tuning of NMDA receptors is therefore critical in regulating excitatory tone, and thereby avoiding toxicity to neurons, as well as in mediating cognitive functions [61]. Pathophysiological changes in the function or expression of NMDA receptors have been implicated in numerous neurodegenerative disorders such as AD, schizophrenia, and epilepsy, as well as in normal physiological processes. NMDA receptors are postsynaptic heteromeric complexes and comprise predominant NR1 and NR2 subtypes, and they are implicated in synaptic transmission and plasticity in the CNS [62]. Ligand-gated ion channel receptors possess a mechanism of activation that presupposes the presence of one or several co-agonists, for example, glycine or D-serine, which modulate their response to synaptic activation. This dual requirement also ensures that NMDA receptors will only be activated in response to high levels of synaptic stimulation, thereby providing a protective barrier against overstimulation on the neural pathways, resulting in something known as excitotoxicity [63]. The last property of NMDA receptors that, in the opinion of the author, should be mentioned is the fact that these receptors possess a voltage-dependent character. This feature is one of those that distinguish NMDA receptors from other types of GLURs. In resting membrane potentials, the receptor and its ion channel are blocked by Mg^2+^ to prevent ion flow [64]. This block is relieved upon depolarization of the postsynaptic neuron, particularly through the activation of the AMPA receptor. When opened, NMDA receptors become permeable to sodium and calcium ions, and allow the potassium out of the cell. The need for this is especially important, since Ca^2+^ is involved as a second messenger that causes intracellular signaling cascades, protein synthesis, and changes in gene expressions that lead to structural and functional synaptic modification [65]. Such an increase in Ca^2+^ concentration is active for synaptic plasticity procedures, including long-term potentiation (LTP) and long-term depression (LTD). LTP, which enhances synaptic connections, is believed to be the cellular basis of learning and memory. However, LTD reduces synaptic connectivity and is implicated in memory formation, as well as in the pruning of redundant connections. The ability of the brain to strengthen communication between neurons is crucial for cognitive flexibility, which is necessary for get used to new information [66]. Additionally, NMDA receptors have several roles in neurodevelopment, and are capable of affecting the processes of neuronal growth and synapse formation during crucial stages in brain maturation. Their malfunction or operational incorrectness contribute to numerous neurological illnesses, for example, AD, schizophrenia, and epilepsy [67]. In particular, the dysregulation of the signaling of NMDA receptors can result in the impaired performance of synaptic transmission and, consequently, cognitive dysfunction in AD. Schizophrenic subjects have available NMDA receptor hypofunction that is linked to the positive and negative symptoms of the disorder. Current studies are focused on evaluating the effectiveness of treatment–NMDA pathway approaches [68]. This information can be used to suggest that manipulating the activity of NMDA receptors might provide therapeutic targets for diseases involving GLU. For example, NMDA receptor antagonists like memantine have been used for symptomatic improvement in dementia associated with AD by giving neuroprotection against excitotoxicity. In conclusion, it can be stated that NMDA receptors do not only function in fast-excitatory transmission, but are also critically involved in synaptic plasticity and cognition. The specific gating mechanisms, ion selectivity, and their roles in intracellular signaling processes also attest to their roles in neuronal survival and functionality. A better grasp of the NMDA receptor’s molecular workings is inherently useful as it relates to the creation of novel NMDA receptor treatment philosophies in cases of neurological illnesses [69].

AMPA receptors can be well described as a type of iGLURs. Tetrameric AMPA receptors are formed from various subtypes, such as GLUA1, GLUA2, GLUA3, and GLUA4, and are important for the high velocity of fundamental excitatory transmission in the brain of humans [70]. These receptors are known to open a cation channel that allows the entry of Na^+^ ions and a small amount of Ca^2+^ ions essential in the production of postsynaptic potentials. The fast kinetics that characterize AMPA receptors mean that neurons are capable of responding to fast synaptic transmission almost immediately [71]. These characteristics position them for multiple cognitive processes, because they contribute to most of the excitatory postsynaptic currents (EPSCs) at glutamatergic synapses. Ca^2+^-dependent facilitation refers to a process where, when GLU is released to the synaptic cleft and binds to the AMPA receptors, it leads to the depolarization of the post-synaptic neuron. This relative depolarization is, however, necessary to spread the excitatory signal through distinct circuits in the neural networks, which is one of the main principles of learning and memory formation, as well as regular neuronal communication [72]. Second, the circuit efficiency and speed at which AMPA receptors provide transmission appropriately contribute to the synaptic plasticity to constantly adapt synaptic connections on a real-time basis, as required by the brain. Consequently, AMPA receptors play a crucial role in the performance of excitatory synapse circuits and play a substantial part in neural mechanisms involved in cognition and behavior. However, with structural similarities to the AMPA receptors, the kainate receptors differ from them pharmacologically, and could be described in terms of their subunit compositions. They favor the entry of the Na^+^ ions and, in some instances, the Ca^2+^ ions that enhance neuronal excitability. Although they are involved in synaptic transmission in part, their general functioning tends to be not as significant as that of AMPA receptors [73].

There is particular interest in the ability of these receptors to release different neurotransmitters; the kainate receptors have been demonstrated to impact the pre-synaptic level. This modulatory function enables kainate receptors to shape the excitatory signaling occurring in neural circuits, which makes them important members of the synaptic regulating apparatus. Through modulating other neurotransmitters, kainate receptors play an important role in the modulation of homeostasis of excitatory/inhibitory balance in global CNS function, which is critical to physiological, cognitive, and neurological processes. These distinctive roles reveal that further clarification is required regarding GLU signaling, which encompasses a wide range of distinct receptor types from a structural perspective and functional handling at synapses [74].

Kainate is one type of metabotropic receptor that binds to GLU and has been categorized into three groups; on the other hand, those featuring bidirectional coupling include G-protein coupled receptors, voltage-gated ion channel receptors, and intracellular calcium receptors. mG receptors are G-protein-coupled receptors that cannot open ion channels, but instead regulate the intracellular signal transduction pathways of second messengers [75]. There are eight known subtypes of mGLURs, classified into three groups based on their signaling pathways [76,77].

mGLUR1 and mGLUR5 belong to Group I metabotropic glutamate receptors, which operate principally postsynaptically and are actively involved in synaptic processes. When bound by glutamate, these receptors induce intracellular events through phospholipase C (PLC). This activation results in the formation of inositol trisphosphate (IP_3_) and diacylglycerol (DAG), thus causing the release of calcium ions from intracellular stores and the activation of Protein kinase C (PKC), respectively [78,79,80]. This signaling pathway is relevant to synaptic function, since Group I mGLURs are implicated in increasing NMDA receptor activity, which leads to LTP and changes to synaptic strength. Through modulating these mechanisms, therefore, Group I mGLURs are involved in synaptic plasticity, with relevance in learning and memory processes as well as in the homeostatic regulation of the optimality of the excitatory signals that underpin neural communication [81].

Primarily found presynaptically, Group II metabotropic glutamate receptors (mGLUR2 and mGLUR3) play an essential regulatory role in synaptic transmission. When activated, these receptors reduce neuronal excitability by inhibiting adenylate cyclase, which lowers cyclic AMP (cAMP) levels [82]. One other way they regulate things is by influencing neurotransmitter release by blocking calcium channels. GLU homeostasis in the brain is maintained by Group II mGLURs, which inhibit excessive synaptic activity. To protect neurons from excitotoxicity and keep synaptic connections stable, they take part in negative feedback systems that limit the excitation of neural circuits. Group II mGLURs contribute to general brain health and play a crucial role in excitatory signal balance via several activities [83,84,85].

The metabotropic glutamate receptors belonging to Group III (mGluR4, mGluR6, mGLUR7, and mGLUR8) are mostly found presynaptically, and are crucial in controlling synaptic transmission [77,86]. These mGLURs, like Group II receptors, lower cAMP levels by inhibiting adenylate cyclase activity. Reduced neural excitability is one effect of this drop in cAMP [87]. Further impacting synaptic signaling, Group III mGLURs impede neurotransmitter release by modifying voltage-gated calcium channels. Group III mGLURs are involved in excitotoxicity protection by modulating synaptic transmission, which in turn helps to avoid cell damage caused by excessive neuronal activity. They aid in preserving the brain’s neuronal health and regulating excitatory neurotransmission across these pathways [88].

Controlling synaptic transmission and plasticity relies on the interaction of two types of GLUreceptors—iGLURs and mGLURs. The modulatory role played by mGLURs leads to variations in synaptic strength over the long term, in contrast to the fast-excitatory responses mediated by iGLURs [89]. Learning, memory, and neural adaptability all rely on this integration [90]. When mGLURs are activated, they may affect the induction of LTP or LTD by either amplifying or dampening the actions of iGLURs. For example, LTP development may be aided by activating Group I mGLURs, which increase NMDA receptor-mediated currents. By controlling intracellular signaling pathways and ion channel activity, mGLURs also control neuronal excitability in general. To keep neural circuits robust and responsive to synaptic activity, this modulation is critical for balancing excitation and inhibition. In a coordinated fashion, iGLURs and mGLURs help the brain’s plasticity and the dynamic character of synaptic connection [91].

The rapid termination of GLU transmission is necessary to avoid excitotoxicity, which results from the overactivation of GLURs. The EAATs on neighboring glial cells, especially astrocytes, and, to a lesser degree, on neurons, are responsible for this. Among the five recognized subtypes of EAAT, the two most abundant in astrocytes are EAAT1 and EAAT2 [92]. When it comes to GLU clearance and recycling, astrocytes are crucial. Astrocytes use the enzyme glutamine synthetase to turn GLU back into GLN once it has been taken up. In this process, GLU is converted to GLN by adding ammonia; the GLN transporters then return the product to the neurons. Avoiding neurotoxicity and keeping excitatory neurotransmission in check, this mechanism is known as the glutamate–glutamine cycle [93].

Normal brain function relies on GLU levels being regulated. Excitotoxicity is one of the various pathological situations that may result from dysregulation, such as defective GLU absorption or excessive release. It is implicated in a number of neurological illnesses, including stroke, epilepsy, and neurodegenerative diseases like Alzheimer’s. Neuronal activity, metabolic states, and the expression of important transporters and enzymes are among the many variables that may affect GLU production, release, and uptake [94]. For instance, GLN absorption and metabolism are both boosted when neuronal activity is high because the demand for GLU is higher. On the other hand, levels of extracellular GLU might rise when astrocytes are unable to rid the cell of glutamate due to factors including oxidative stress or energy shortage [95]. Various receptors targeted as therapeutic targets in AD treatment and management are summarized in Table 1.

## 4. Glutamate Dysregulation in Alzheimer’s Disease

GLU is the main excitatory neurotransmitter in the CNS, and it acts on both metabotropic and ionotropic receptors [57]. It is situated at the intersection of several metabolic pathways, and is crucial for memory and learning processes. The loss of synapses and neuronal death in AD impairs glutamatergic neuron activity, which can affect memory, cognition, and behavior, including cortical and hippocampus processing [26,107,108].

### 4.1. Excitotoxicity and Neurodegeneration

GLU signaling dysregulation is a major contributor to excitotoxicity and neurotoxicity in AD [109]. An excessive influx of Ca^2+^ ions into neurons is caused by one of the main processes, which is the chronic overactivation of NMDA receptors. Several cellular activities, such as synaptic plasticity and neurotransmitter release, rely on Ca^2+^ under typical circumstances [95]. However dangerous quantities of intracellular calcium build up when NMDA receptors are continually engaged. This excess sets off a cascade of detrimental intracellular processes. Several calcium-dependent enzymes, including calpains, protein kinases, and phospholipases, are activated by the high calcium concentration [110]. These enzymes set in motion processes that lead to the production of reactive oxygen species (ROS). An imbalance between free radical production and the body’s detoxification capabilities causes oxidative stress, which in turn is caused by ROS formation. Damage to lipids, proteins, and DNA caused by oxidative stress results in genetic mutations, decreased protein function, and a breakdown of cell membrane integrity. The viability and health of neurons may be severely compromised by this kind of injury. The continued elevation of intracellular Ca^2+^ interferes with mitochondrial activity and causes oxidative stress as well. The dysfunction of mitochondria, which are essential for ATP generation, reduces the amount of energy available to neurons, which further impairs their function [110]. Programmed cell death pathways may be activated when impaired mitochondria produce pro-apoptotic proteins. Cellular damage is worsened and neuronal death is contributed to by the vicious cycle that is created when oxidative stress and mitochondrial malfunction converge [111]. Furthermore, the excitotoxicity caused by GLU dysregulation impacts not only specific neurons, but also the communication across different brain circuits, and the synaptic connections between them. Cognitive decline, memory impairment, and the general course of AD are all caused by the loss of synaptic connections, which occurs when neurons deteriorate and die. Therapeutic approaches that target GLU signaling may be able to save neurons and maintain cognitive function, since this multimodal effect emphasizes the crucial role of GLU dysregulation in the pathogenesis of Alzheimer’s [112,113,114].

### 4.2. Amyloid-β and Tau Interaction with Glutamate Signaling

**Figure 6:**
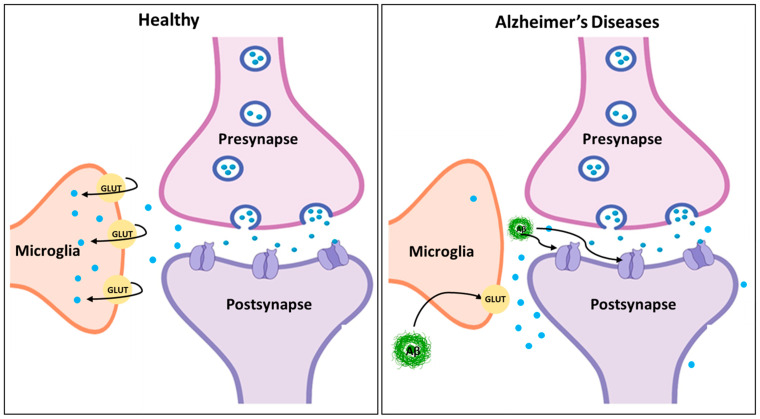
The diagram illustrates synaptic transmission under healthy and Alzheimer’s disease (AD) conditions. In the healthy synapse (**left** panel), glutamate (GLUT) is released from the presynapse into the synaptic cleft, where it binds to postsynaptic receptors, facilitating normal synaptic signaling. Microglia regulate extracellular glutamate levels by efficient uptake through glutamate transporters, maintaining synaptic homeostasis. In Alzheimer’s disease (**right** panel), amyloid-beta (Aβ) aggregates disrupt synaptic function by impairing glutamate uptake by microglia and promoting excitotoxicity. Aβ also interacts with synaptic receptors, contributing to postsynaptic dysfunction. These pathological changes highlight the impaired glutamate regulation and neurotoxicity characteristic of AD. The concept of the figure was adopted from [123] and has been modified and recreated on PowerPoint.

The disruption of glutamatergic neurotransmission is a major function of amyloid-β (Aβ) plaques and tau tangles in AD, which worsens excitotoxicity and adds to synaptic failure. It is well-known that Aβ enhances the overactivation of NMDA receptors, which results in an overabundance of calcium influx and heightened excitotoxicity [115,116,117]. This persistent stimulation not only hinders cognitive abilities by promoting neuronal death, but also by interfering with synaptic plasticity. In addition, Aβ may make presynaptic neurons release more GLU and astrocytes absorb less of it, leading to higher amounts of GLU outside of cells, as shown in Figure 6, which can have excitotoxic consequences [118]. Alternatively, tau disease disrupts mGLUR signaling, a process crucial for controlling synaptic transmission and plasticity. Intracellular signaling pathways may be altered when tau undergoes abnormal hyperphosphorylation, which disrupts its normal interactions with cellular components [119,120]. Because of this interference, mGLURs are no longer able to protect neurons from excitotoxicity by efficiently modulating synaptic activity. A toxic environment is created by the combined effects of Aβ-induced NMDA receptor overactivation and the tau-mediated impairment of mGLUR signaling [121]. This environment promotes neurodegeneration, and ultimately contributes to the cognitive decline characteristic of Alzheimer’s disease by undermining the brain’s capacity for plasticity and adaptation [122].

### 4.3. Impairment of Synaptic Plasticity and Memory

Synaptic plasticity processes, including learning and memory-related LTP, rely on glutamate. As a result of abnormalities in GLU transmission, LTP is severely impaired in AD, preventing the body from responding to experience by strengthening synaptic connections [103,124]. Synaptic modulation is essential for information encoding and memory consolidation, but this impairment reduces this ability [125,126]. Excitotoxicity, which occurs when NMDA receptors are overactivated as a result of high GLU levels, may harm neurons and cause synapses to break down. This strongly correlates with the cognitive impairments seen in Alzheimer’s patients, and hinders their capacity to create new memories and recall old ones [127]. The impairment of synaptic plasticity is further intensified by the malfunctioning of mGLURs. The total efficiency of synaptic transmission is reduced when mGLURs fail to efficiently regulate synaptic activity, which disrupts the balance between excitation and inhibition [76,128]. A decline in the adaptive capacity of the neural networks involved in memory formation makes it harder for people with AD to learn new things and remember what they already know. This series of events emphasizes how important **GLU** transmission is for brain function and how detrimental its dysregulation is to the development of AD [129].

## 5. Therapeutic Targeting of Glutamate Signaling in Alzheimer’s Disease

### 5.1. NMDA Receptor Modulators

Ionotropic glutamate receptors known as NMDA receptors have special characteristics that allow them to play important roles in practically every facet of both healthy and diseased brain activities. NDMA receptors are cation-selective ligand-gated ion channels [130,131]. Allosteric modulation, competitive agonists or antagonists, and channel blockers can all be used to control NMDA receptor channel activity. In an effort to determine how NMDA receptor subtypes are involved in both healthy and pathological circumstances, subunit select modulators would be helpful [132,133,134].

#### 5.1.1. Memantine

Using NMDA receptor antagonists, in particular, to therapeutically target GLU signaling in AD has shown promise [135]. For this reason, memantine, an NMDA receptor antagonist with a low affinity, was created, and is now used to treat mild to severe AD [136]. It preserves the normal synaptic transmission crucial for cognitive function while specifically blocking the abnormal overactivation of NMDA receptors, according to its unique pharmacological profile. Increased intracellular calcium levels may activate harmful signaling pathways in AD, which is driven by the chronic overactivation of NMDA receptors caused by excessive GLU release [137,138,139]. Cognitive impairment is accompanied by neuronal death and synaptic malfunction, both of which are caused by this excitotoxicity [140]. By binding to NMDA receptors less strongly than conventional antagonists, memantine circumvents this problem. Consequently, it selectively inhibits receptor activation under physiologically normal circumstances while permitting excessive activation to occur in the presence of high GLU levels. Important for learning and memory, this quality aids in maintaining synaptic plasticity and regular neurotransmission. Several important components make up memantine’s mode of action [141]. Memantine decreases neurodegenerative oxidative stress and mitochondrial dysfunction by blocking excessive calcium influx. Furthermore, it has the potential to stabilize receptor activation, which improves synaptic signaling; this, in turn, may improve cognitive performance and delay the advancement of AD symptoms. There is some evidence that memantine helps enhance cognitive function, DL tasks, and quality of life. Potentially adding to memantine’s therapeutic advantages are suggestions that it may have neuroprotective properties [142,143,144,145]. Combining memantine with additional treatments that target various parts of GLU signaling or other neurotransmitter systems may increase its efficacy and provide more complete treatment methods; this is an area of active investigation. To summarize, memantine is a huge step forward in the pharmacological treatment of AD since it targets GLU dysregulation, a major disease mechanism. A significant treatment approach in the continuous endeavor to counteract the cognitive deficits associated with AD, it mitigates excitotoxicity while maintaining normal neural activity [142].

#### 5.1.2. Potential Novel NMDA Antagonists

More selective chemicals that target individual NMDA receptor subunits are the subject of research into possible new NMDA antagonists. Obtaining improved treatment results with less adverse effects using non-selective NMDA antagonists, such as memantine, is the goal of this strategy. Researchers want to improve the effectiveness of controlling AD symptoms by developing antagonists that specifically bind to certain receptor subtypes [146]. This will allow them to fine-tune the regulation of glutamatergic transmission. Several benefits may be derivable from selective NMDA antagonists. For example, it has been shown that the NR2B subunit is essential for synaptic plasticity and memory formation [147]. Potentially improving cognitive functions without compromising synaptic transmission, antagonists that target NR2B-containing receptors may provide neuroprotection without compromising normal NMDA receptor function. Furthermore, these new compounds may lessen the possibility of side effects such as drowsiness or psychotomimetic symptoms, which are often linked to wider NMDA receptor blocking, by sparing other receptor subtypes [148]. The structural and functional features of NMDA receptor subunits are also the subject of continuing studies into the identification of particular binding sites for novel antagonists. Other neurodegenerative diseases marked by GLU dysregulation may also be amenable to this precision medicine strategy, which is already being used to develop more tailored treatments for AD. There is optimism in the prospect of more effective and more tolerated therapies for a variety of disorders, including AD, as these new NMDA antagonists go through preclinical and clinical studies [148].

### 5.2. Modulation of mGluRs

#### 5.2.1. mGluR2/3 Agonists

There is new evidence for the restoration of neuronal function through the modulation of the mGLUR2 and mGLUR3 in AD. The modulators of this receptor play a crucial role in reducing glutamate release from presynaptic neurons, thereby mitigating excitotoxicity—a key mechanism contributing to the neuronal damage and cognitive decline characteristic of the disease. mGLUR2 and mGLUR3 are predominantly localized on presynaptic terminals, where they act as inhibitory receptors within the glutamatergic system, regulating synaptic glutamate levels and preventing overactivation [149]. Depending on the activating substances, these receptors block the adenylate cyclase, thus decreasing the amount of cAMP. This ultimately lowers the release of all neurotransmitters, such as glutamate, which is useful in owing the distinctive overproduction of excitatory signals in AD [150]. This is particularly important because excessive glutamate release is often seen in several neurological disorders, and mGluR2/3 agonists can thus indirectly reduce glutamatergic hyperactivity, prevent excitotoxic neuronal death, and therefore promote both structural and functional synaptic plasticity [151]. Many experimental animal studies have indicated that aversive effects of mGLUR2/3 agonists exist that improve neuronal transmission and ameliorate cognitive impairment. Besides preventing neurons from being destroyed by excitotoxicity, the decrease in GLU release may help enhance synaptic plasticity and other memory functions that have been impaired in AD. At the moment, there are several active studies seeking to determine the roles of drug mGluR2/3 agonists as they affect people with AD. These trials will focus on evaluating these compounds for efficacy, rate, and side effects, as well as any positive impact on cognition at different stages of progression. Initial studies have been promising; the agonists should enhance cognition and may halt the disease’s course through the regulation of GLU. In conclusion, new pharmacologic approaches for modulating glutamate signaling in AD are represented by mGLUR2/3 agonists. On these grounds, these compounds may help in diminishing excitotoxicity and providing a better synaptic milieu that can indeed improve the cognition and quality of life of patients suffering from this dreaded disease. With future discoveries, mGLUR2/3 agonists will be incorporated into the line of medications that are utilized for the treatment of AD [152].

#### 5.2.2. mGluR5 Antagonists

In this context, the mGluR5 antagonists are expected to receive increasing amounts of attention, especially with criticisms of Aβ-induced excitotoxicity, which are being implicated in AD. Investigations have also shown that in Alzheimer’s, there is an increased density in mGluR5 receptors that makes neurons more susceptible to excitotoxic injury. These compounds may block the pathogenic effects of Aβ on neuronal activity, and antagonism against mGluR5 may counteract the excitotoxic changes in synapses leading to neurodegenerative processes [153]. In basic animal studies, it has been seen that mGluR5 antagonists reduce Aβ-mediated excitotoxicity, increase synaptic plasticity, and improve cognitive function in animal models [153]. Some of them are currently undergoing experimentation into grasping the effectiveness and tolerability of these antagonists in human beings—the primary purpose being to see whether these antagonists could act as a tool in shielding the neurons and preserving the mental abilities of patients diagnosed with AD. Similarly, the other existing and potential therapeutic targeting approaches act on GLU transporters, especially EAATs. The EAAT2 protein, located on the membrane of astrocytes, plays a crucial role in clearing glutamate from the synapse. Among the five glutamate transporters (EAAT1-5), EAAT2 is particularly important for maintaining synaptic transmission and preventing excitotoxicity in neurons. The dysfunction of EAAT2 has been linked to AD. When EAAT2 function is impaired, excess glutamate builds up in the synaptic cleft, overstimulating postsynaptic glutamatergic receptors. This overstimulation causes an influx of Na^+^ and Ca^2+^ ions into neurons, leading to excitotoxicity. This process triggers apoptotic or necrotic pathways, contributing to the neuronal damage and degeneration seen in AD [154,155,156].

These transporters play a critical role in facilitating the removal of unwanted GLU from the synaptic cleft to avoid excitotoxicity while maintaining a normal level of neurotransmitters. The promotion of the expression or function of EAATs might be a potential strategy for improving the elimination of GLU in the brain. This strategy regards the enhancement of GLU uptake into neighboring glial cells and neurons to prevent the excitotoxicity arising from high extracellular concentrations of GLU. Thus far, efforts are being made to discover molecules that will increase EAAT levels or improve the efficiency of their transport. These may include medication, genetic treatments that tend to alter the levels of these carriers, or even an individual’s diet. This research reveals that enhancing EAAT activity results in better synaptic and neurorestorative function; therefore, this path offers a potential avenue for the therapeutic management of AD. Thus, the dual inhibition of mGluR5 and the adjustment of EAAT function can be regarded as an integrated strategy in treating the multifaceted problem of GLU dysfunction in AD [106,157]. Because excitotoxicity is minimized through a range of mechanisms, these approaches can be seen to have the potential for neuronal preservation, the maintenance of synapses, and an improvement in the general cognitive functionality of those affected. These therapeutic leads might offer a more progressive means of treating AD as research continues. A summary of various potential therapeutic strategies is listed in Table 2, with their targets, mechanisms and current clinical statuses, and the challenges associate with them.

## 6. Glutamate Signaling in the Diagnosis of AD

Biomarkers are important in AD identification and the outcome assessment of therapeutic approaches, especially in those involving GLU signaling pathways [163]. Subsequently, the NIA-AA introduced the AT(N) framework to characterize research based on Aβ deposition, pathologic tau, and neurodegeneration biomarkers [164]. The diagnosis of AD mainly relies on imaging biomarkers including structural MRI (sMRI) and positron emission tomography (PET) [165,166]. Structural MRI shows hippocampal loss, whilst 18FDG-PET shows diminished glucose uptake may also be relevant to GLU-mediated synaptic deficit [167]. Aβ deposition detected by Amyloid PET mediates NMDA receptor overactivation and excitotoxicity, while tau PET detects neurofibrillary tangles that disrupt mGluR signaling, implying tau-mediated synaptic dysfunction. Furthermore, in addition to imaging, biochemical markers derived from CSF including Aβ42, phosphorylated tau (P-tau181), and neurofilament light chain (NfL) suggest first-tier indicators of AD pathophysiology and neuronal degeneration that are associated with the disorder of GLU signaling [168]. Some recent innovations in blood biomarkers such as P-tau 217 and GFAP provide opportunities for monitoring progression and neuroinflammation, respectively [169]. These biomarkers are necessary for the assessment of GLU therapies, for instance, NMDA receptor antagonists including memantine or mGluR by means of evaluation of the alterations of the synaptic function and neurodegeneration [97,170].

Nevertheless, problems still exist; for example, several biomarkers cannot distinguish AD from other diseases with similar patterns of neuropathological changes, and the CSF and imaging data can be discordant [171,172]. However, biomarkers have a great potential capacity to enhance the diagnostics and therapy of AD under the conditions of further fine-tuning the biomarker’s accuracy, as well as the application of a personalized medicine concept [173].

## 7. Clinical Drugs

Previous medicines for AD are mainly composed of cholinesterase inhibitors and NMDA receptor antagonists that act on neurotransmitter abnormalities but not on disease progression [174]. Donepezil, rivastigmine and galantamine, being examples of AChEIs, help in increasing the levels of acetylcholine, which improves cognitive and behavioral aspects [175,176]. Donepezil is available as an oral and transdermal preparation, with the transdermal form being approved in 2022 due to its lesser gastrointestinal side effects and greater ease of administration [177,178]. Rivastigmine and galantamine have better pharmacokinetics too; however, in the context of combining these inhibitors together or with other neurologic drugs, antioxidants or metal chelators are being investigated to enhance efficacy and safety profiles [179,180,181]. Memantine is an NMDA receptor antagonist that reduces the GLU signaling in excitotoxicity; hence, it is effective for mild to moderate to severe stages of AD. Namzaric is another alternative for treating AD because of the memantine–donepezil single-tablet regimen that is available, especially for stage III and IV patients. Still, these drugs could not change mechanisms that cause the disease at the same time [30,182]. Allophysics, including sodium oligomannate (GV-971), work on the mechanism of neuroinflammation by modulating gut microbiota, and have potential to decrease amyloidosis and neuroinflammation in animal models [183,184]. Sodium oligomannate is still in phase IV clinical trial [185]. Aducanumab and other monoclonal antibodies targeting Aβ show moderate efficacy in the reduction in amyloid burden and cognitive decline, with risks associated with ARIA [186,187,188]. Moreover, brexpiprazole, which was initially approved for treating psychiatric disorders, can be effective in managing AD-associated agitated patients [189]. Due to the expense and low probability of success of remedies being developed for AD, new efforts in drug repurposing have emerged, especially with the use of AI to determine existing drugs that have potential neuroprotective effects [190,191,192]. This approach is particularly attractive because the compounds submitted for approval are likely to have well-established safety profiles, and are likely to be producible at lower cost, as they were initially designed for other diseases [190]. On the subject of AD, the clinical trials also give an overview of the pharmacotherapeutic targets available for the management of the condition through neurotransmitter modification, Aβ lowering, tau protein stabilization, and inflammation moderation. Most of these mechanisms also have functions in GLU signaling pathways, an important area of study in AD, because the over-stimulation of NMDA receptors in glutamatergic pathways is linked to excitotoxicity, which is involved in neuronal and cognitive dysfunction [193,194]. GLU involvement in AD has received considerable interest because of its involvement in synaptic plasticity, learning, and memory. Inappropriately high concentrations of GLU cause the overstimulation of the NMDA receptor, which promotes neurotoxicity, an influx of calcium, and mitochondrial disorder. Clinical trials on NMDA receptor antagonists including memantine have already shown their modest efficacy due to the protection of neurons against excitotoxic effects in AD patients [97]. The need to target GLU signaling is well substantiated by research being done on such molecules as Varoglutamstat (PQ912), which targets glutaminyl cyclase and thereby decreases the generation of pyroglutamate-modified Aβ, one of the most pathogenic forms of the protein. Such comorbidity of Aβ and GLU indicates that drugs targeting both the amyloid and glutamatergic systems should provide synergistic neuroprotection [195,196]. However, the Aβ hypothesis continues to be the major focus of AD therapeutic intervention, although new developments in the disease indicate that this is not a single-symptom disease, and that other pathways such as tau aggregation, neuroinflammation, and neurotransmitter imbalance also need to be targeted. This aligns with the consideration of GLU as a putative therapeutic target of the disease because of its involvement in excitotoxicity, tau hyperphosphorylation, and synaptic loss. The exact treatments targeting tau consist of Tau aggregation inhibitors such as TRx0237 (LMTX) [197,198,199] and Bepranemab [200], which implies that those exclusively dealing with amyloid or tau plus the GLU pathway may be a more successful strategy. In conclusion, prospective and registered clinical trials indicate the need for various anti-AD therapeutic approaches targeting the pathological process. In the case of GLU signaling, small-molecule drugs or modulators of the NMDA receptors and related excitotoxic pathways appear to be fruitful approaches. There is now a need to envisage how these pathways could be integrated into other therapeutic strategies, possibly with other avenues presently being pursued through amyloid and tau-directed actions, and to design better, disease-modifying interventions for AD. All this comes at the best time, with the contemporary focus on precision medicine, particularly concerning NDDs, such as Alzheimer’s, wherein multiple disease mechanisms need to be targeted at the same time.

## 8. Limitations and Challenges

The therapeutic targeting of GLU signaling in AD harbors several issues and difficulties. A major issue frequently reported by researchers concerns bias-related issues of off-target effect. Since GLU is involved in essentially every form of learning, memory, and synaptic plasticity critical to brain function [201,202], it is challenging to modulate its signaling pathways without engendering dysfunction in the brain. Due to the high level of engagement of the GLU in the process of transmitting excitation, the approaches that are used to address this neurotransmitter often interfere with normal receptors, and this results in side effects such as coordinating ability damage or tremors [203]. Another significant problem is AD stage dependency. The nature and severity of impairments in AD change over time (regarding each stage) and the efficiency of interventions aimed at modifying GLU signaling can also be dependent on the stage of the disease. Although it is believed that the correction of GLU dysregulation through early intervention would be effective in clients by virtue of minimizing both excitotoxicity and synapse destruction, the process of ascertaining those with early-stage AD is quite challenging because of the absence of unique biomarkers. Consequently, the majority of the patient populace may potentially receive no utility from these treatments until the disease has advanced beyond the ability of GLU modulation to make a positive difference [204,205]. The basic disturbance of GLU is only a fraction of the entire picture [206]. Simultaneously, glutamate receptor-targeting medications are used to treat behavioral disorders, stabilize or temporarily improve cognitive skills, and slow the progression of disease. These treatments assist AD patients and their caregivers in maintaining their independence and enhancing their quality of life, despite the fact that they are not curative. Nevertheless, these therapies only address the effects of AD rather than its underlying cause, and their efficacy is at best limited and transient. Before the neurodegenerative process starts, these medication treatments could be more helpful in the early asymptomatic period. The limited efficiency of these treatments is also attributed to other factors, such as the challenge of brain drug targeting because of the blood–brain barrier (BBB) restricting passage from the circulation to the central nervous system. Permeability problems at the BBB in AD are a major reason why many medication studies fail [207].

## 9. Future Directions and Prospects

Directions concerning the targeting of GLU signaling for AD in the future are concerned with the complexity and uniqueness of existing approaches. One potentially more fruitful area of development involves the incorporation of biomarkers and the use of targeted genetic and proteomic signatures in an attempt at the more accurate categorization of patient population characteristics. This approach could enable the development of more specific GLU-based treatments for those patients who are most likely to benefit from intervention in this pathway. Since AD is a disease with many factors and mechanisms, including Aβ deposition, neurofibrillary tangles, and inflammation, to name but a few, targeting GLU only might not be effective enough. Adding these other agents to the GLU modulators could form a more comprehensive approach to treatment. Thus, the co-administration of NMDA receptor antagonists with agents that decrease Aβ accumulation or with anti-inflammatory drugs could be more effective in decelerating the disease course than using the drugs alone [208]. However, there is a continuous potential for to enhance drug delivery systems’ specificity and the effectiveness of the therapies that target GLU. Another great challenge facing the treatment of neurodegenerative diseases is the inability to supply the brain with drugs due to the biological barrier of the BBB [209,210]. Improved drugs that have better permeability through the BBB include nanoparticles such as liposome, antibody–drug conjugates, or other nanomaterials [211,212,213] that can help deliver the GLU modulators to the brain efficiently while lowering the drug’s impact on the rest of the body. These developed drug delivery systems may also be used for the sustained release of the medications so as to allow a steady regulation of GLU, thus enhancing patient’s performance. Finally, future treatments of AD related to GLU would necessarily include more specific and also multi-potency treatment strategies facilitated by advanced drug delivery systems that can enhance the efficacy and safety of the therapies [214,215]. These innovations could facilitate future successful interventions, with benefits related to the prevention of the progression of AD.

## 10. Conclusions

Glutamate has diverse functions in the development of AD: it modulates synaptic plasticity and excitotoxicity, and affects neuronal survival. This is why disturbances in the same neurotransmitter, namely, GLU, are considered one of the critical factors leading to neurodegeneration in AD; such pathophysiological changes include the overactivation of NMDA receptors as well as the impairment of GLU clearance. Such disruptions are responsible for the characteristic cognitive impairments and memory loss evident in the AD patients that make signaling through GLU worth targeting. Currently available medicines, such as memantine, that work by inhibiting excitotoxicity can manage a few aspects of the disease, but they have failed to slow the progression of the disease. Furthermore, enhancing the specificity of GLU-based interventional approaches poses limitations; for example, off-target effects, efficacy issues across different phases of the disease, and the heterogeneity of AD. Employing GLU alone seems not to be sufficient for all patients because AD is polyetiopathogenic, involving Aβ-plaques, tau pathology, neuroinflammation, and vascular elements. It is recommended that in the future, more investigations should be conducted on molecular- and biomarker-guided therapy, and targeted diagnostic tools based on the GLU pathway have been developed to address patients’ needs. Such a patient-tailored treatment plan correlated with the knowledge of individual patient’s genomes could be combined with the concept of using combination therapies and advanced drug delivery systems, which could potentially provide patient-oriented approaches that are faster, safer, and more effective. Combination with GLU modulatory agents can be used in conjunction with Aβ, tau, and anti-inflammatory therapeutic interventions to achieve a better therapeutic outcome and potentially slow down the dynamics of the disease. In conclusion, studies of GLU signaling in AD have achieved certain progress; however, to develop further therapies for this disease, researchers should focus on more complex mechanisms in combination with the expected outcomes of the effective drug. In the course of future investigations, there is potential for these strategies to not only aid in the reduction in symptoms, but also to help elucidate the mechanisms that cause the diseases to progress, consequently helping patients with AD to achieve better results and a better quality of life.

## Figures and Tables

**Table 1:** A summary of different types of receptors with their functions.

Receptor Type	Dysregulation in Alzheimer’s Disease (AD)	Therapeutic Targeting	Reference
NMDA Receptors	- Over-activation leads to excitotoxicity - Increased Ca^2+^ influx leads to mitochondrial damage	- Memantine (NMDA antagonist) selectively blocks pathological activation while preserving normal signaling	[96,97,98,99]
AMPA Receptors	- Reduced function impairs synaptic plasticity and cognitive functions	- Emerging research on enhancing AMPA receptor signaling to restore synaptic function in AD	[100,101]
Kainate Receptors	- Lesser known role in AD but implicated in modulating glutamatergic excitability	- No current specific AD-targeted therapies involving kainate receptors	[102]
mGluR1/5 (Group I)	- Tau pathology disrupts mGLUR5 signaling - Enhances excitotoxicity in AD	- mGLUR5 antagonists under investigation for reducing Aβ-induced excitotoxicity	[103]
mGluR2/3 (Group II)	- Loss of function contributes to increased glutamate release and excitotoxicity	- mGLUR2/3 agonists are being tested for reducing glutamate release and neuroprotection in AD	[104,105]
EAAT Transporters	- Reduced function in AD leads to accumulation of extracellular glutamate, increasing excitotoxicity	- Strategies to enhance EAAT function under investigation to improve glutamate clearance and reduce toxicity	[106]

**Table 2:** Summary of therapeutic strategies for Alzheimer’s disease management by targeting glutamate receptors, transporters, and enzymes involved in glutamate metabolism.

Therapeutic Strategy	Target/Mechanism	Clinical Status	Challenges/Limitations	References
NMDA Receptor Antagonists	Blocks pathological overactivation of NMDA receptors, reducing excitotoxicity without disrupting normal signaling	Memantine (FDA-approved for moderate-to-severe AD)	Limited efficacy in advanced stages of AD; potential off-target effects on normal neurotransmission	[96,97,158]
Selective NMDA Subunit Antagonists	Targets specific NMDA receptor subunits (e.g., NR2B) to enhance selectivity and reduce side effectsGluN2B subunit-containing NMDARs preventing cognitive deficits in early AD	In preclinical and early clinical trials	High specificity is required to avoid interference with essential NMDA receptor functions	[159]
mGluR5 Antagonists	Reduces Aβ-induced excitotoxicity by inhibiting Group I mGLURs (mGluR5)	In early-stage clinical trials for AD	Potential for disrupting normal mGluR5-mediated plasticity and learning	[153]
mGluR2/3 Agonists	Activates Group II mGLURs to inhibit GLU release, reducing excitotoxicity and neuronal damage	In clinical trials for neuroprotection in AD	Limited understanding of long-term effects on synaptic transmission	[104]
EAAT Enhancers	Enhances the function of EAAT transporters to clear excess GLU from the synaptic cleft	Experimental phase, preclinical research	Difficulty in delivering EAAT-enhancing compounds across the blood–brain barrier	[106]
Glutaminase Inhibitors	Reduces GLU synthesis by inhibiting the conversion of GLN to GLU	Investigational, in preclinical trials for neurodegenerative diseases	Potential disruption of essential GLU-dependent brain functions	[160]
Gene Therapy Approaches	Targets genes involved in the GLU metabolism or receptor regulation (e.g., increasing EAAT expression)	Experimental, early-stage research	Ethical and technical challenges in gene therapy; long-term safety not yet established	[161,162]

## Data Availability

Not applicable.

## References

[1] WHO (2022). A Blueprint for Dementia Research.

[2] Knopman D.S., Amieva H., Petersen R.C., Chételat G., Holtzman D.M., Hyman B.T., Nixon R.A., Jones D.T. (2021). Alzheimer disease. Nat. Rev. Dis. Prim..

[3] Puranik N., Yadav D., Song M. (2023). Advancements in the Application of Nanomedicine in Alzheimer’s Disease: A Therapeutic Perspective. Int. J. Mol. Sci..

[4] Di Benedetto G., Burgaletto C., Bellanca C.M., Munafò A., Bernardini R., Cantarella G. (2022). Role of Microglia and Astrocytes in Alzheimer’s Disease: From Neuroinflammation to Ca^2+^ Homeostasis Dysregulation. Cells.

[5] Fakhoury M. (2018). Microglia and Astrocytes in Alzheimer’s Disease: Implications for Therapy. Curr. Neuropharmacol..

[6] Singh D. (2022). Astrocytic and microglial cells as the modulators of neuroinflammation in Alzheimer’s disease. J. Neuroinflammation.

[7] Cummings J.L., Osse A.M.L., Kinney J.W. (2023). Alzheimer’s Disease: Novel Targets and Investigational Drugs for Disease Modification. Drugs.

[8] Gabr M.T., Ibrahim M.M. (2019). Multitarget therapeutic strategies for Alzheimer’s disease. Neural Regen. Res..

[9] Querfurth H.W., LaFerla F.M. (2010). Alzheimer’s Disease. N. Engl. J. Med..

[10] Castillo-Vazquez S.K., Massieu L., Rincón-Heredia R., la Torre P.G.-D., Quiroz-Baez R., Gomez-Verjan J.C., Rivero-Segura N.A. (2024). Glutamatergic Neurotransmission in Aging and Neurodegenerative Diseases: A Potential Target to Improve Cognitive Impairment in Aging. Arch. Med Res..

[11] Kandimalla R., Reddy P.H. (2017). Therapeutics of Neurotransmitters in Alzheimer’s Disease. J. Alzheimer’s Dis..

[12] Du X., Li J., Li M., Yang X., Qi Z., Xu B., Liu W., Xu Z., Deng Y. (2020). Research progress on the role of type I vesicular glutamate transporter (VGLUT1) in nervous system diseases. Cell Biosci..

[13] Zhou Y., Danbolt N.C. (2014). Glutamate as a neurotransmitter in the healthy brain. J. Neural Transm..

[14] (2024). 2024 Alzheimer’s disease facts and figures. Alzheimer’s Dement..

[15] Abdelnour C., Agosta F., Bozzali M., Fougère B., Iwata A., Nilforooshan R., Takada L.T., Viñuela F., Traber M. (2022). Perspectives and challenges in patient stratification in Alzheimer’s disease. Alzheimer’s Res. Ther..

[16] Crabbé M., Dirkx N., Casteels C., Van Laere K. (2019). Excitotoxic neurodegeneration is associated with a focal decrease in metabotropic glutamate receptor type 5 availability: An in vivo PET imaging study. Sci. Rep..

[17] Hynd M.R., Scott H.L., Dodd P.R. (2004). Glutamate-mediated excitotoxicity and neurodegeneration in Alzheimer’s disease. Neurochem. Int..

[18] Gupta K., Hardingham G.E., Chandran S. (2013). NMDA receptor-dependent glutamate excitotoxicity in human embryonic stem cell-derived neurons. Neurosci. Lett..

[19] Olloquequi J., Cornejo-Córdova E., Verdaguer E., Soriano F.X., Binvignat O., Auladell C., Camins A. (2018). Excitotoxicity in the pathogenesis of neurological and psychiatric disorders: Therapeutic implications. J. Psychopharmacol..

[20] Anastacio H.T.D., Matosin N., Ooi L. (2022). Neuronal hyperexcitability in Alzheimer’s disease: What are the drivers behind this aberrant phenotype?. Transl. Psychiatry.

[21] Cummings J. (2021). New approaches to symptomatic treatments for Alzheimer’s disease. Mol. Neurodegener..

[22] Graff-Radford J., Yong K.X.X., Apostolova L.G., Bouwman F.H., Carrillo M., Dickerson B.C., Rabinovici G.D., Schott J.M., Jones D.T., Murray M.E. (2021). New insights into atypical Alzheimer’s disease in the era of biomarkers. Lancet. Neurol..

[23] Gasiorowska A., Wydrych M., Drapich P., Zadrozny M., Steczkowska M., Niewiadomski W., Niewiadomska G. (2021). The Biology and Pathobiology of Glutamatergic, Cholinergic, and Dopaminergic Signaling in the Aging Brain. Front. Aging Neurosci..

[24] Falgàs N., Walsh C.M., Neylan T.C., Grinberg L.T. (2021). Deepen into sleep and wake patterns across Alzheimer’s disease phenotypes. Alzheimer’s Dement..

[25] Guo T., Zhang D., Zeng Y., Huang T.Y., Xu H., Zhao Y. (2020). Molecular and cellular mechanisms underlying the pathogenesis of Alzheimer’s disease. Mol. Neurodegener..

[26] Bukke V.N., Archana M., Villani R., Romano A.D., Wawrzyniak A., Balawender K., Orkisz S., Beggiato S., Serviddio G., Cassano T. (2020). The Dual Role of Glutamatergic Neurotransmission in Alzheimer’s Disease: From Pathophysiology to Pharmacotherapy. Int. J. Mol. Sci..

[27] Uddin S., Al Mamun A., Kabir T., Ashraf G.M., Bin-Jumah M.N., Abdel-Daim M.M. (2021). Multi-Target Drug Candidates for Multifactorial Alzheimer’s Disease: AChE and NMDAR as Molecular Targets. Mol. Neurobiol..

[28] Devanand D.P., Fremont R. (2024). Cognitive Enhancers and Treatments for Alzheimer’s Disease. Tasman’s Psychiatry.

[29] Cheong S.L., Tiew J.K., Fong Y.H., Leong H.W., Chan Y.M., Chan Z.L., Kong E.W.J. (2022). Current Pharmacotherapy and Multi-Target Approaches for Alzheimer’s Disease. Pharmaceuticals.

[30] Kabir T., Uddin S., Al Mamun A., Jeandet P., Aleya L., Mansouri R.A., Ashraf G.M., Mathew B., Bin-Jumah M.N., Abdel-Daim M.M. (2020). Combination Drug Therapy for the Management of Alzheimer’s Disease. Int. J. Mol. Sci..

[31] Hung S.-Y., Fu W.-M. (2017). Drug candidates in clinical trials for Alzheimer’s disease. J. Biomed. Sci..

[32] Abdallah A.E. (2024). Review on anti-alzheimer drug development: Approaches, challenges and perspectives. RSC Adv..

[33] Atri A. (2019). The Alzheimer’s Disease Clinical Spectrum: Diagnosis and Management. Med. Clin..

[34] Maciejewska K., Czarnecka K., Szymański P. (2021). A review of the mechanisms underlying selected comorbidities in Alzheimer’s disease. Pharmacol. Rep..

[35] Schousboe A., Scafidi S., Bak L.K., Waagepetersen H.S., McKenna M.C. (2014). Glutamate Metabolism in the Brain Focusing on Astrocytes. Adv. Neurobiol..

[36] Andersen J.V., Markussen K.H., Jakobsen E., Schousboe A., Waagepetersen H.S., Rosenberg P.A., Aldana B.I. (2021). Glutamate metabolism and recycling at the excitatory synapse in health and neurodegeneration. Neuropharmacology.

[37] Cuellar-Santoyo A.O., Ruiz-Rodríguez V.M., Mares-Barbosa T.B., Patrón-Soberano A., Howe A.G., Portales-Pérez D.P., Graf A.M., Estrada-Sánchez A.M. (2023). Revealing the contribution of astrocytes to glutamatergic neuronal transmission. Front. Cell. Neurosci..

[38] Ding L., Xu X., Li C., Wang Y., Xia X., Zheng J.C. (2020). Glutaminase in microglia: A novel regulator of neuroinflammation. Brain, Behav. Immun..

[39] Katabathula S., Davis P.B., Xu R. (2023). Comorbidity-driven multi-modal subtype analysis in mild cognitive impairment of Alzheimer’s disease. Alzheimer’s Dement..

[40] Zhang D., Hua Z., Li Z. (2024). The role of glutamate and glutamine metabolism and related transporters in nerve cells. CNS Neurosci. Ther..

[41] Gong X., Guo R., Li X., Yang Y., Lin W. (2023). A red-emitting mitochondria targetable fluorescent probe for detecting viscosity in HeLa, zebrafish, and mice. Anal. Methods.

[42] Wang S., Wang L., Qin X., Turdi S., Sun D., Culver B., Reiter R.J., Wang X., Zhou H., Ren J. (2020). ALDH2 contributes to melatonin-induced protection against APP/PS1 mutation-prompted cardiac anomalies through cGAS-STING-TBK1-mediated regulation of mitophagy. Signal Transduct. Target. Ther..

[43] Salasova A., Monti G., Andersen O.M., Nykjaer A. (2022). Finding memo: Versatile interactions of the VPS10p-Domain receptors in Alzheimer’s disease. Mol. Neurodegener..

[44] Andersen J.V., Schousboe A. (2023). Milestone Review: Metabolic dynamics of glutamate and GABA mediated neurotransmission—The essential roles of astrocytes. J. Neurochem..

[45] Marde V.S., Atkare U.A., Gawali S.V., Tiwari P.L., Badole S.P., Wankhede N.L., Taksande B.G., Upaganlawar A.B., Umekar M.J., Kale M.B. (2021). Alzheimer’s disease and sleep disorders: Insights into the possible disease connections and the potential therapeutic targets. Asian J. Psychiatry.

[46] Fehsel K., Christl J. (2022). Comorbidity of osteoporosis and Alzheimer’s disease: Is ‘AKT’-ing on cellular glucose uptake the missing link?. Ageing Res. Rev..

[47] Errasti-Murugarren E., Palacín M. (2021). Heteromeric Amino Acid Transporters in Brain: From Physiology to Pathology. Neurochem. Res..

[48] Stobart J.L., Anderson C.M. (2013). Multifunctional role of astrocytes as gatekeepers of neuronal energy supply. Front. Cell. Neurosci..

[49] Mahmoud S., Gharagozloo M., Simard C., Gris D. (2019). Astrocytes Maintain Glutamate Homeostasis in the CNS by Controlling the Balance between Glutamate Uptake and Release. Cells.

[50] Ullah G. (2019). The role of transporters and synaptic cleft morphology in glutamate and GABA homeostasis and their effect on neuronal function. bioRxiv.

[51] Gunes S., Aizawa Y., Sugashi T., Sugimoto M., Rodrigues P.P. (2022). Biomarkers for Alzheimer’s Disease in the Current State: A Narrative Review. Int. J. Mol. Sci..

[52] Reiner A., Levitz J. (2018). Glutamatergic Signaling in the Central Nervous System: Ionotropic and Metabotropic Receptors in Concert. Neuron.

[53] Brown P.M.G.E., Dawe G.B., Feltz A., Bowie D. (2020). Structural and Functional Properties of Ionotropic and Metabotropic Glutamate Receptors. Physiology of Neurons.

[54] Yadav P., Podia M., Kumari S.P., Mani I. (2023). Glutamate receptor endocytosis and signaling in neurological conditions. Prog. Mol. Biol. Transl. Sci..

[55] Song T., Song X., Zhu C., Patrick R., Skurla M., Santangelo I., Green M., Harper D., Ren B., Forester B.P. (2021). Mitochondrial dysfunction, oxidative stress, neuroinflammation, and metabolic alterations in the progression of Alzheimer’s disease: A meta-analysis of in vivo magnetic resonance spectroscopy studies. Ageing Res. Rev..

[56] Ragnarsson L., Dodd P.R., Latif M.R. (2022). Role of Ionotropic Glutamate Receptors in Neurodegenerative and Other Disorders. Handbook of Neurotoxicity.

[57] Roman J.Y.M., González C.C. (2024). Glutamate and excitotoxicity in central nervous system disorders: Ionotropic glutamate receptors as a target for neuroprotection. Neuroprotection.

[58] Lloret A., Esteve D., Lloret M.-A., Cervera-Ferri A., Lopez B., Nepomuceno M., Monllor P. (2019). When Does Alzheimer′s Disease Really Start? The Role of Biomarkers. Int. J. Mol. Sci..

[59] Porsteinsson A.P., Isaacson R.S., Knox S., Sabbagh M.N., Rubino I. (2021). Diagnosis of Early Alzheimer’s Disease: Clinical Practice in 2021. J. Prev. Alzheimer’s Dis..

[60] Nedelec T., Couvy-Duchesne B., Monnet F., Daly T., Ansart M., Gantzer L., Lekens B., Epelbaum S., Dufouil C., Durrleman S. (2022). Identifying health conditions associated with Alzheimer’s disease up to 15 years before diagnosis: An agnostic study of French and British health records. Lancet Digit. Health.

[61] Zhang X.X., Tian Y., Wang Z.T., Ma Y.H., Tan L., Yu J.T. (2021). The Epidemiology of Alzheimer’s Disease Modifiable Risk Factors and Prevention. J. Prev. Alzheimer’s Dis..

[62] Logroscino G. (2020). Prevention of Alzheimer’s disease and dementia: The evidence is out there, but new high-quality studies and implementation are needed. J. Neurol. Neurosurg. Psychiatry.

[63] Crous-Bou M., Minguillón C., Gramunt N., Molinuevo J.L. (2017). Alzheimer’s disease prevention: From Risk factors to early intervention. Alzheimer’s Res. Ther..

[64] Omura J.D., McGuire L.C., Patel R., Baumgart M., Lamb R., Jeffers E.M., Olivari B.S., Croft J.B., Thomas C.W., Hacker K. (2022). Modifiable Risk Factors for Alzheimer Disease and Related Dementias Among Adults Aged ≥45 Years—United States, 2019. Mmwr-Morbidity Mortal. Wkly. Rep..

[65] Zhang D.-F., Li M. (2023). Toward a Full Understanding of Causal and Modifiable Risk Factors for Alzheimer’s Disease by Integrative Phenome-wide Association Studies. Biol. Psychiatry.

[66] Silva M.V.F., Loures C.d.M.G., Alves L.C.V., de Souza L.C., Borges K.B.G., Carvalho M.d.G. (2019). Alzheimer’s disease: Risk factors and potentially protective measures. J. Biomed. Sci..

[67] Beata B.-K., Wojciech J., Johannes K., Piotr L., Barbara M. (2023). Alzheimer’s Disease—Biochemical and Psychological Background for Diagnosis and Treatment. Int. J. Mol. Sci..

[68] Thakral S., Yadav A., Singh V., Kumar M., Kumar P., Narang R., Sudhakar K., Verma A., Khalilullah H., Jaremko M. (2023). Alzheimer’s disease: Molecular aspects and treatment opportunities using herbal drugs. Ageing Res. Rev..

[69] Stanciu G.D., Luca A., Rusu R.N., Bild V., Chiriac S.I.B., Solcan C., Bild W., Ababei D.C. (2020). Alzheimer’s Disease Pharmacotherapy in Relation to Cholinergic System Involvement. Biomolecules.

[70] Hampel H., Lista S., Khachaturian Z.S. (2012). Development of biomarkers to chart all Alzheimer’s disease stages: The royal road to cutting the therapeutic Gordian Knot. Alzheimer’s Dement..

[71] Sutphen C.L., Fagan A.M., Holtzman D.M. (2014). Progress Update: Fluid and Imaging Biomarkers in Alzheimer’s Disease. Biol. Psychiatry.

[72] Lista S., Garaci F.G., Ewers M., Teipel S., Zetterberg H., Blennow K., Hampel H. (2013). CSF Aβ1-42 combined with neuroimaging biomarkers in the early detection, diagnosis and prediction of Alzheimer’s disease. Alzheimer’s Dement..

[73] Reiman E.M. (2017). Putting AD treatments and biomarkers to the test. Nat. Rev. Neurol..

[74] Nair J.D., Wilkinson K.A., Henley J.M., Mellor J.R. (2021). Kainate receptors and synaptic plasticity. Neuropharmacology.

[75] Qizilbash N., Whitehead A., Higgins J., Wilcock G., Schneider L., Farlow M. (1998). Cholinesterase Inhibition for Alzheimer Disease: A Meta-analysis of the Tacrine Trials. JAMA.

[76] Bodzęta A., Berger F., MacGillavry H.D. (2022). Subsynaptic mobility of presynaptic mGluR types is differentially regulated by intra- and extracellular interactions. Mol. Biol. Cell.

[77] Huang L., Xiao W., Wang Y., Li J., Gong J., Tu E., Long L., Xiao B., Yan X., Wan L. (2023). Metabotropic glutamate receptors (mGluRs) in epileptogenesis: An update on abnormal mGluRs signaling and its therapeutic implications. Neural Regen. Res..

[78] Jarrott B. (2017). Tacrine: In vivo veritas. Pharmacol. Res..

[79] Mango D., Ledonne A. (2023). Updates on the Physiopathology of Group I Metabotropic Glutamate Receptors (mGluRI)-Dependent Long-Term Depression. Cells.

[80] Su L.-D., Wang N., Han J., Shen Y. (2021). Group 1 Metabotropic Glutamate Receptors in Neurological and Psychiatric Diseases: Mechanisms and Prospective. Neuroscientist.

[81] Watkins P.B., Zimmerman H.J., Knapp M.J., Gracon S.I., Lewis K.W. (1994). Hepatotoxic Effects of Tacrine Administration in Patients with Alzheimer’s Disease. JAMA.

[82] Ríos C.d.L., Marco-Contelles J. (2019). Tacrines for Alzheimer’s disease therapy. III. The PyridoTacrines. Eur. J. Med. Chem..

[83] Birks J.S., Harvey R.J. (2018). Donepezil for dementia due to Alzheimer’s disease. Cochrane Database Syst. Rev..

[84] Bocchio M., Lukacs I.P., Stacey R., Plaha P., Apostolopoulos V., Livermore L., Sen A., Ansorge O., Gillies M.J., Somogyi P. (2019). Group II metabotropic glutamate receptors mediate presynaptic inhibition of excitatory transmission in pyramidal neurons of the human cerebral cortex. Front. Cell. Neurosci..

[85] Trepanier C., Lei G., Xie Y.-F., MacDonald J.F. (2013). Group II metabotropic glutamate receptors modify N-methyl-D-aspartate receptors via Src kinase. Sci. Rep..

[86] Dasgupta A., Lim Y.J., Kumar K., Baby N., Pang K.L.K., Benoy A., Behnisch T., Sajikumar S., Neurobiology S.K.L.O.M. (2020). China Group III metabotropic glutamate receptors gate long-term potentiation and synaptic tagging/capture in rat hippocampal area CA2. eLife.

[87] Cui X., Guo Y.-E., Fang J.-H., Shi C.-J., Suo N., Zhang R., Xie X. (2019). Donepezil, a drug for Alzheimer’s disease, promotes oligodendrocyte generation and remyelination. Acta Pharmacol. Sin..

[88] Brewster J.T., Dell’Acqua S., Thach D.Q., Sessler J.L. (2019). Classics in Chemical Neuroscience: Donepezil. ACS Chem. Neurosci..

[89] Crupi R., Impellizzeri D., Cuzzocrea S. (2019). Role of Metabotropic Glutamate Receptors in Neurological Disorders. Front. Mol. Neurosci..

[90] Feldman H.H., Lane R. (2007). Rivastigmine: A placebo controlled trial of twice daily and three times daily regimens in patients with Alzheimer’s disease. J. Neurol. Neurosurg. Psychiatry.

[91] Rösler M., Anand R., Cicin-Sain A., Gauthier S., Agid Y., Dal-Bianco P., Stähelin H.B., Hartman R., Gharabawi M., Bayer T. (1999). Efficacy and safety of rivastigmine in patients with Alzheimer’s disease: International randomised controlled trial Commentary: Another piece of the Alzheimer’s jigsaw. BMJ.

[92] Coyle J., Kershaw P. (2001). Galantamine, a cholinesterase inhibitor that allosterically modulates nicotinic receptors: Effects on the course of Alzheimer’s disease. Biol. Psychiatry.

[93] Scott L.J., Goa K.L. (2000). Galantamine: A review of its use in Alzheimer’s disease. Drugs.

[94] Marco-Contelles J., do Carmo Carreiras M., Rodríguez C., Villarroya M., García A.G. (2006). Synthesis and pharmacology of Galantamine. Chem. Rev..

[95] Robinson D.M., Keating G.M. (2006). Memantine: A review of its use in Alzheimer’s disease. Drugs.

[96] Siddiqui A.J., Badraoui R., Jahan S., Alshahrani M.M., Siddiqui M.A., Khan A., Adnan M. (2023). Targeting NMDA receptor in Alzheimer’s disease: Identifying novel inhibitors using computational approaches. Front. Pharmacol..

[97] Liu J., Chang L., Song Y., Li H., Wu Y. (2019). The Role of NMDA Receptors in Alzheimer’s Disease. Front. Neurosci..

[98] Zhang Y., Li P., Feng J., Wu M. (2016). Dysfunction of NMDA receptors in Alzheimer’s disease. Neurol. Sci..

[99] Wang R., Reddy P.H. (2017). Role of Glutamate and NMDA Receptors in Alzheimer’s Disease. J. Alzheimer’s Dis..

[100] Babaei P. (2021). NMDA and AMPA receptors dysregulation in Alzheimer’s disease. Eur. J. Pharmacol..

[101] Ning L., Shen R., Xie B., Jiang Y., Geng X., Dong W. (2024). AMPA receptors in Alzheimer disease: Pathological changes and potential therapeutic targets. J. Neuropathol. Exp. Neurol..

[102] Pellegrini-Giampietro D., Bennett M., Zukin R. (1994). Ampa/kainate receptor gene expression in normal and alzheimer’s disease hippocampus. Neuroscience.

[103] Srivastava A., Das B., Yao A.Y., Yan R. (2020). Metabotropic Glutamate Receptors in Alzheimer’s Disease Synaptic Dysfunction: Therapeutic Opportunities and Hope for the Future. J. Alzheimer’s Dis..

[104] Li S.H., Abd-Elrahman K.S., Ferguson S.S. (2022). Targeting mGluR2/3 for treatment of neurodegenerative and neuropsychiatric diseases. Pharmacol. Ther..

[105] Abd-Elrahman K.S., Sarasija S., Ferguson S.S. (2023). The Role of Neuroglial Metabotropic Glutamate Receptors in Alzheimer’s Disease. Curr. Neuropharmacol..

[106] Wood O.W.G., Yeung J.H.Y., Faull R.L.M., Kwakowsky A. (2022). EAAT2 as a therapeutic research target in Alzheimer’s disease: A systematic review. Front. Neurosci..

[107] Cassano T., Serviddio G., Gaetani S., Romano A., Dipasquale P., Cianci S., Bellanti F., Laconca L., Romano A.D., Padalino I. (2011). Glutamatergic alterations and mitochondrial impairment in a murine model of Alzheimer disease. Neurobiol. Aging.

[108] Rupsingh R., Borrie M., Smith M., Wells J., Bartha R. (2011). Reduced hippocampal glutamate in Alzheimer disease. Neurobiol. Aging.

[109] Zott B., Konnerth A. (2023). Impairments of glutamatergic synaptic transmission in Alzheimer’s disease. Semin. Cell Dev. Biol..

[110] Reisberg B., Doody R., Stöffler A., Schmitt F., Ferris S., Möbius H.J. (2003). Memantine in Moderate-to-Severe Alzheimer’s Disease. New Engl. J. Med..

[111] Zong Y., Li H., Liao P., Chen L., Pan Y., Zheng Y., Zhang C., Liu D., Zheng M., Gao J. (2024). Mitochondrial dysfunction: Mechanisms and advances in therapy. Signal Transduct. Target. Ther..

[112] Greig S.L. (2015). Memantine ER/Donepezil: A Review in Alzheimer’s Disease. CNS Drugs.

[113] Lewerenz J., Maher P. (2015). Chronic glutamate toxicity in neurodegenerative diseases-What is the evidence?. Front Neurosci.

[114] Maestú F., de Haan W., Busche M.A., DeFelipe J. (2021). Neuronal excitation/inhibition imbalance: Core element of a translational perspective on Alzheimer pathophysiology. Ageing Res. Rev..

[115] Deardorff W.J., Grossberg G.T. (2016). A fixed-dose combination of memantine extended-release and donepezil in the treatment of moderate-to-severe Alzheimer’s disease. Drug Des. Dev. Ther..

[116] Rudy C.C., Hunsberger H.C., Weitzner D.S., Reed M.N. (2015). The Role of the Tripartite Glutamatergic Synapse in the Pathophysiology of Alzheimer’s Disease. Aging Dis..

[117] Danysz W., Parsons C.G. (2012). Alzheimer’s disease, β-amyloid, glutamate, NMDA receptors and memantine-Searching for the connections. Br. J. Pharmacol..

[118] Talantova M., Sanz-Blasco S., Zhang X., Xia P., Akhtar M.W., Okamoto S.I., Dziewczapolski G., Nakamura T., Cao G., Pratt A.E. (2013). Aβ induces astrocytic glutamate release, extrasynaptic NMDA receptor activation, and synaptic loss. Proc. Natl. Acad. Sci. USA.

[119] Benek O., Korabecny J., Soukup O. (2020). A Perspective on Multi-target Drugs for Alzheimer’s Disease. Trends Pharmacol. Sci..

[120] Benarroch E.E. (2018). Glutamatergic synaptic plasticity and dysfunction in Alzheimer disease: Emerging mechanisms. Neurology.

[121] Rajmohan R., Reddy P.H. (2017). Amyloid-Beta and Phosphorylated Tau Accumulations Cause Abnormalities at Synapses of Alzheimer’s disease Neurons. J. Alzheimer’s Dis..

[122] Syed Y.Y. (2020). Sodium Oligomannate: First Approval. Drugs.

[123] Fairless R., Bading H., Diem R. (2021). Pathophysiological Ionotropic Glutamate Signalling in Neuroinflammatory Disease as a Therapeutic Target. Front. Neurosci..

[124] Citri A., Malenka R.C. (2008). Synaptic Plasticity: Multiple Forms, Functions, and Mechanisms. Neuropsychopharmacology.

[125] Wang T., Kuang W., Chen W., Xu W., Zhang L., Li Y., Li H., Peng Y., Chen Y., Wang B. (2020). A phase II randomized trial of sodium oligomannate in Alzheimer’s dementia. Alzheimer’s Res. Ther..

[126] Luboeinski J., Tetzlaff C. (2021). Memory consolidation and improvement by synaptic tagging and capture in recurrent neural networks. Commun. Biol..

[127] Dong X.-X., Wang Y., Qin Z.-H. (2009). Molecular mechanisms of excitotoxicity and their relevance to pathogenesis of neurodegenerative diseases. Acta Pharmacol. Sin..

[128] Bellone C., Lüscher C., Mameli M. (2008). Mechanisms of synaptic depression triggered by metabotropic glutamate receptors. Cell. Mol. Life Sci..

[129] Xiao S., Chan P., Wang T., Hong Z., Wang S., Kuang W., He J., Pan X., Zhou Y., Ji Y. (2021). A 36-week multicenter, randomized, double-blind, placebo-controlled, parallel-group, phase 3 clinical trial of sodium oligomannate for mild-to-moderate Alzheimer’s dementia. Alzheimer’s Res. Ther..

[130] Hansen K.B., Yi F., Perszyk R.E., Menniti F.S., Traynelis S.F. (2017). NMDA receptors in the central nervous system. Methods Mol. Biol..

[131] Hanson J.E., Yuan H., Perszyk R.E., Banke T.G., Xing H., Tsai M.-C., Menniti F.S., Traynelis S.F. (2023). Therapeutic potential of N-methyl-D-aspartate receptor modulators in psychiatry. Neuropsychopharmacology.

[132] Chen H., Dong Y., Wu Y., Yi F. (2023). Targeting NMDA receptor signaling for therapeutic intervention in brain disorders. Prog. Neurobiol..

[133] Hansen K.B., Yi F., Perszyk R.E., Furukawa H., Wollmuth L.P., Gibb A.J., Traynelis S.F. (2018). Structure, function, and allosteric modulation of NMDA receptors. J. Gen. Physiol..

[134] Monaghan D.T., Irvine M.W., Costa B.M., Fang G., Jane D.E. (2012). Pharmacological modulation of NMDA receptor activity and the advent of negative and positive allosteric modulators. Neurochem. Int..

[135] Cummings J., Aisen P., Apostolova L.G., Atri A., Salloway S., Weiner M. (2021). Aducanumab: Appropriate Use Recommendations. J. Prev. Alzheimer’s Dis..

[136] Tang B.C., Wang Y.T., Ren J. (2023). Basic information about memantine and its treatment of Alzheimer’s disease and other clinical applications. Ibrain.

[137] Dhillon S. (2021). Aducanumab: First Approval. Drugs.

[138] Olivares D., Deshpande V.K., Shi Y., Lahiri D.K., Greig N.H., Rogers J.T., Huang X. (2012). N-Methyl D-Aspartate (NMDA) Receptor Antagonists and Memantine Treatment for Alzheimer’s Disease, Vascular Dementia and Parkinson’s Disease. Curr. Alzheimer Res..

[139] Tampi R.R., van Dyck C.H. (2007). Memantine: Efficacy and safety in mild-to-severe Alzheimer’s disease. Neuropsychiatr. Dis. Treat..

[140] Tamm L.N., Miller R. (2024). NMDA Receptors in Stroke: Pathways and Potential Treatments. J. Undergrad. Res..

[141] Behl T., Kaur I., Sehgal A., Singh S., Sharma N., Makeen H.A., Albratty M., Alhazmi H.A., Felemban S.G., Alsubayiel A.M. (2022). “Aducanumab” making a comeback in Alzheimer’s disease: An old wine in a new bottle. Biomedicine & Pharmacotherapy.

[142] Larkin H.D. (2023). Lecanemab Gains FDA Approval for Early Alzheimer Disease. JAMA.

[143] Folch J., Busquets O., Ettcheto M., Sánchez-López E., Castro-Torres R.D., Verdaguer E., Garcia M.L., Olloquequi J., Casadesús G., Beas-Zarate C. (2018). Memantine for the Treatment of Dementia: A Review on its Current and Future Applications. J. Alzheimer’s Dis..

[144] Tari P.K., Parsons C.G., Collingridge G.L., Rammes G. (2023). Memantine: Updating a rare success story in pro-cognitive therapeutics. Neuropharmacology.

[145] Dominguez E., Chin T.-Y., Chen C.-P., Wu T.-Y. (2011). Management of moderate to severe Alzheimer’s disease: Focus on memantine. Taiwan. J. Obstet. Gynecol..

[146] Harris E. (2023). Alzheimer Drug Lecanemab Gains Traditional FDA Approval. JAMA.

[147] Zeng B.-S.H., Tseng P.-T., Liang C.-S. (2023). Lecanemab in Early Alzheimer’s Disease. N. Engl. J. Med..

[148] Sims J.R., Zimmer J.A., Evans C.D., Lu M., Ardayfio P., Sparks J., Wessels A.M., Shcherbinin S., Wang H., Nery E.S.M. (2023). Donanemab in Early Symptomatic Alzheimer Disease: The TRAILBLAZER-ALZ 2 Randomized Clinical Trial. JAMA.

[149] Hippius H., Neundörfer G. (2003). The discovery of Alzheimer’s disease. Dialog-Clin. Neurosci..

[150] Hajjo R., Sabbah D.A., Abusara O.H., Al Bawab A.Q. (2022). A Review of the Recent Advances in Alzheimer’s Disease Research and the Utilization of Network Biology Approaches for Prioritizing Diagnostics and Therapeutics. Diagnostics.

[151] Burns S., Selman A., Sehar U., Rawat P., Reddy A.P., Reddy P.H. (2022). Therapeutics of Alzheimer’s Disease: Recent Developments. Antioxidants.

[152] Jin L.E., Wang M., Galvin V.C., Lightbourne T.C., Conn P.J., Arnsten A.F., Paspalas C.D. (2017). mGluR2 versus mGluR3 Metabotropic Glutamate Receptors in Primate Dorsolateral Prefrontal Cortex: Postsynaptic mGluR3 Strengthen Working Memory Networks. Cereb. Cortex.

[153] Kumar A., Dhull D.K., Mishra P.S. (2015). Therapeutic potential of mGluR5 targeting in Alzheimer’s disease. Front. Neurosci..

[154] Thal D.R. (2002). Excitatory amino acid transporter EAAT-2 in tangle-bearing neurons in Alzheimer’s disease. Brain Pathol..

[155] Manisha C., Selvaraj A., Jubie S., Nanjan C.M.J., Joghee N.M., Clement J.P., Justin A. (2020). Positive allosteric activation of glial EAAT-2 transporter protein: A novel strategy for Alzheimer’s disease. Med. Hypotheses.

[156] O’donovan S.M., Sullivan C.R., McCullumsmith R.E. (2017). The role of glutamate transporters in the pathophysiology of neuropsychiatric disorders. Npj Schizophr..

[157] Takahashi K., Foster J.B., Lin C.-L.G. (2015). Glutamate transporter EAAT2: Regulation, function, and potential as a therapeutic target for neurological and psychiatric disease. Cell. Mol. Life Sci..

[158] Kabir T., Sufian M.A., Uddin S., Begum M.M., Akhter S., Islam A., Mathew B., Islam S., Amran S., Ashraf G.M. (2019). NMDA Receptor Antagonists: Repositioning of Memantine as a Multitargeting Agent for Alzheimer’s Therapy. Curr. Pharm. Des..

[159] Hu N.-W., Klyubin I., Anwyl R., Rowan M.J. (2009). GluN2B subunit-containing NMDA receptor antagonists prevent Aβ-mediated synaptic plasticity disruption in vivo. Proc. Natl. Acad. Sci. USA.

[160] Bayraktar A., Li X., Kim W., Zhang C., Turkez H., Shoaie S., Mardinoglu A. (2023). Drug repositioning targeting glutaminase reveals drug candidates for the treatment of Alzheimer’s disease patients. J. Transl. Med..

[161] Loera-Valencia R., Piras A., Ismail M.A.M., Manchanda S., Eyjolfsdottir H., Saido T.C., Johansson J., Eriksdotter M., Winblad B., Nilsson P. (2018). Targeting Alzheimer’s disease with gene and cell therapies. J. Intern. Med..

[162] Takahashi K., Kong Q., Lin Y., Stouffer N., Schulte D.A., Lai L., Liu Q., Chang L.-C., Dominguez S., Xing X. (2015). Restored glial glutamate transporter EAAT2 function as a potential therapeutic approach for Alzheimer’s disease. J. Exp. Med..

[163] Bhole R.P., Chikhale R.V., Rathi K.M. (2024). Current biomarkers and treatment strategies in Alzheimer disease: An overview and future perspectives. IBRO Neurosci. Rep..

[164] Jack C.R., Bennett D.A., Blennow K., Carrillo M.C., Dunn B., Haeberlein S.B., Holtzman D.M., Jagust W., Jessen F., Karlawish J. (2018). NIA-AA Research Framework: Toward a biological definition of Alzheimer’s disease. Alzheimer Dement..

[165] Márquez F., Yassa M.A. (2019). Neuroimaging Biomarkers for Alzheimer’s Disease. Mol. Neurodegener..

[166] Young P.N.E., Estarellas M., Coomans E., Srikrishna M., Beaumont H., Maass A., Venkataraman A.V., Lissaman R., Jiménez D., Betts M.J. (2020). Imaging biomarkers in neurodegeneration: Current and future practices. Alzheimer’s Res. Ther..

[167] van Oostveen W.M., de Lange E.C.M. (2021). Imaging Techniques in Alzheimer’s Disease: A Review of Applications in Early Diagnosis and Longitudinal Monitoring. Int. J. Mol. Sci..

[168] Zhang Y., Chen H., Li R., Sterling K., Song W. (2023). Amyloid β-based therapy for Alzheimer’s disease: Challenges, successes and future. Signal Transduct. Target. Ther..

[169] Huber H., Huber H., Blennow K., Blennow K., Zetterberg H., Zetterberg H., Boada M., Boada M., Jeromin A., Jeromin A. (2024). Biomarkers of Alzheimer’s disease and neurodegeneration in dried blood spots—A new collection method for remote settings. Alzheimer’s Dement..

[170] Gautam D., Naik U.P., Naik M.U., Yadav S.K., Chaurasia R.N., Dash D. (2023). Glutamate Receptor Dysregulation and Platelet Glutamate Dynamics in Alzheimer’s and Parkinson’s Diseases: Insights into Current Medications. Biomolecules.

[171] De Ninno G., Giuffrè G.M., Urbani A., Baroni S. (2024). Current perspectives on Alzheimer’s disease fluid biomarkers and future challenges: A narrative review. J. Lab. Precis. Med..

[172] Khoury R., Ghossoub E. (2019). Diagnostic biomarkers of Alzheimer’s disease: A state-of-the-art review. Biomark. Neuropsychiatry.

[173] Qiu S., Cai Y., Yao H., Lin C., Xie Y., Tang S., Zhang A. (2023). Small molecule metabolites: Discovery of biomarkers and therapeutic targets. Signal Transduct. Target. Ther..

[174] Zhang J., Zhang Y., Wang J., Xia Y., Zhang J., Chen L. (2024). Recent advances in Alzheimer’s disease: Mechanisms, clinical trials and new drug development strategies. Signal Transduct. Target. Ther..

[175] Singh B., Day C.M., Abdella S., Garg S. (2024). Alzheimer’s disease current therapies, novel drug delivery systems and future directions for better disease management. J. Control. Release.

[176] Marucci G., Buccioni M., Ben D.D., Lambertucci C., Volpini R., Amenta F. (2021). Efficacy of acetylcholinesterase inhibitors in Alzheimer’s disease. Neuropharmacology.

[177] Dinh L., Lee S., Abuzar S.M., Park H., Hwang S.-J. (2022). Formulation, Preparation, Characterization, and Evaluation of Dicarboxylic Ionic Liquid Donepezil Transdermal Patches. Pharmaceutics.

[178] Buck A., Rezaei K., Quazi A., Goldmeier G., Silverglate B., Grossberg G.T. (2024). The donepezil transdermal system for the treatment of patients with mild, moderate, or severe Alzheimer’s disease: A critical review. Expert Rev. Neurother..

[179] Siddique Y.H., Naz F., Rahul, Varshney H. (2022). Comparative study of rivastigmine and galantamine on the transgenic Drosophila model of Alzheimer’s disease. Curr. Res. Pharmacol. Drug Discov..

[180] Miculas D.C., Negru P.A., Bungau S.G., Behl T., Hassan S.S.U., Tit D.M. (2022). Pharmacotherapy Evolution in Alzheimer’s Disease: Current Framework and Relevant Directions. Cells.

[181] Dighe S.N., De la Mora E., Chan S., Kantham S., McColl G., Miles J.A., Veliyath S.K., Sreenivas B.Y., Nassar Z.D., Silman I. (2019). Rivastigmine and metabolite analogues with putative Alzheimer’s disease-modifying properties in a Caenorhabditis elegans model. Commun. Chem..

[182] Guo J., Wang Z., Liu R., Huang Y., Zhang N., Zhang R. (2020). Memantine, Donepezil, or Combination Therapy—What is the best therapy for Alzheimer’s Disease? A Network Meta-Analysis. Brain Behav..

[183] Wang X., Sun G., Feng T., Zhang J., Huang X., Wang T., Xie Z., Chu X., Yang J., Wang H. (2019). Sodium oligomannate therapeutically remodels gut microbiota and suppresses gut bacterial amino acids-shaped neuroinflammation to inhibit Alzheimer’s disease progression. Cell Res..

[184] Bosch M.E., Dodiya H.B., Michalkiewicz J., Lee C., Shaik S.M., Weigle I.Q., Zhang C., Osborn J., Nambiar A., Patel P. (2024). Sodium oligomannate alters gut microbiota, reduces cerebral amyloidosis and reactive microglia in a sex-specific manner. Mol. Neurodegener..

[185] Huang L.-K., Kuan Y.-C., Lin H.-W., Hu C.-J. (2023). Clinical trials of new drugs for Alzheimer disease: A 2020–2023 update. J. Biomed. Sci..

[186] Haddad H.W., Malone G.W., Comardelle N.J., Degueure A.E., Kaye A.M., Kaye A.D. (2022). Aducanumab, a Novel Anti-Amyloid Monoclonal Antibody, for the Treatment of Alzheimer’s Disease: A Comprehensive Review. Health Psychol. Res..

[187] Shi M., Chu F., Zhu F., Zhu J. (2022). Impact of Anti-amyloid-beta Monoclonal Antibodies on the Pathology and Clinical Profile of Alzheimer’s Disease: A Focus on Aducanumab and Lecanemab. Front Aging Neurosci.

[188] Cummings J., Osse A.M.L., Cammann D., Powell J., Chen J. (2023). Anti-Amyloid Monoclonal Antibodies for the Treatment of Alzheimer’s Disease. BioDrugs.

[189] Lee D., Slomkowski M., Hefting N., Chen D., Larsen K.G., Kohegyi E., Hobart M., Cummings J.L., Grossberg G.T. (2023). Brexpiprazole for the Treatment of Agitation in Alzheimer Dementia. JAMA Neurol..

[190] Cheng F., Wang F., Tang J., Zhou Y., Fu Z., Zhang P., Haines J.L., Leverenz J.B., Gan L., Hu J. (2024). Artificial intelligence and open science in discovery of disease-modifying medicines for Alzheimer’s disease. Cell Rep. Med..

[191] Wu T., Lin R., Cui P., Yong J., Yu H., Li Z. (2024). Deep learning-based drug screening for the discovery of potential therapeutic agents for Alzheimer’s disease. J. Pharm. Anal..

[192] Patwekar M., Patwekar F., Shaikh D., Fatema S.R., Aher S.J., Sharma R. (2023). Receptor-based approaches and therapeutic targets in Alzheimer’s disease along with role of AI in drug designing: Unraveling pathologies and advancing treatment strategies. Appl. Chem. Eng..

[193] Lukiw W.J. (2012). Amyloid beta (Aβ) peptide modulators and other current treatment strategies for Alzheimer’s disease (AD). Expert Opin. Emerg. Drugs.

[194] Xiao D., Zhang C. (2024). Current therapeutics for Alzheimer’s disease and clinical trials. Open Explor..

[195] Hoffmann T., Rahfeld J.-U., Schenk M., Ponath F., Makioka K., Hutter-Paier B., Lues I., Lemere C.A., Schilling S. (2021). Combination of the Glutaminyl Cyclase Inhibitor PQ912 (Varoglutamstat) and the Murine Monoclonal Antibody PBD-C06 (m6) Shows Additive Effects on Brain Aβ Pathology in Transgenic Mice. Int. J. Mol. Sci..

[196] Vijverberg E.G.B., Axelsen T.M., Bihlet A.R., Henriksen K., Weber F., Fuchs K., Harrison J.E., Kühn-Wache K., Alexandersen P., Prins N.D. (2021). Rationale and study design of a randomized, placebo-controlled, double-blind phase 2b trial to evaluate efficacy, safety, and tolerability of an oral glutaminyl cyclase inhibitor varoglutamstat (PQ912) in study participants with MCI and mild AD—VIVIAD. Alzheimer’s Res. Ther..

[197] Panza F., Seripa D., Solfrizzi V., Imbimbo B.P., Santamato A., Lozupone M., Capozzo R., Prete C., Pilotto A., Greco A. (2015). Tau aggregation inhibitors: The future of Alzheimer’s pharmacotherapy?. Expert Opin. Pharmacother..

[198] Cummings J.L., Gonzalez M.I., Pritchard M.C., May P.C., Toledo-Sherman L.M., Harris G.A. (2023). The therapeutic landscape of tauopathies: Challenges and prospects. Alzheimer’s Res. Ther..

[199] Huang L.-K., Chao S.-P., Hu C.-J. (2020). Clinical trials of new drugs for Alzheimer disease. J. Biomed. Sci..

[200] Panza F., Solfrizzi V., Daniele A., Lozupone M. (2023). Passive tau-based immunotherapy for tauopathies. Handb. Clin. Neurol..

[201] Chen T.-S., Huang T.-H., Lai M.-C., Huang C.-W. (2023). The Role of Glutamate Receptors in Epilepsy. Biomedicines.

[202] Pal M.M. (2021). Glutamate: The Master Neurotransmitter and Its Implications in Chronic Stress and Mood Disorders. Front. Hum. Neurosci..

[203] Hoffmann J., Charles A. (2018). Glutamate and Its Receptors as Therapeutic Targets for Migraine. Neurotherapeutics.

[204] Conway M.E. (2020). Alzheimer’s disease: Targeting the glutamatergic system. Biogerontology.

[205] Pinky P.D., Pfitzer J.C., Senfeld J., Hong H., Bhattacharya S., Suppiramaniam V., Qureshi I., Reed M.N. (2022). Recent Insights on Glutamatergic Dysfunction in Alzheimer’s Disease and Therapeutic Implications. Neuroscientist.

[206] Desjardins P., Du T., Jiang W., Peng L., Butterworth R.F. (2012). Pathogenesis of hepatic encephalopathy and brain edema in acute liver failure: Role of glutamine redefined. Neurochem. Int..

[207] Passeri E., Elkhoury K., Morsink M., Broersen K., Linder M., Tamayol A., Malaplate C., Yen F.T., Arab-Tehrany E. (2022). Alzheimer’s Disease: Treatment Strategies and Their Limitations. Int. J. Mol. Sci..

[208] Companys-Alemany J., Turcu A.L., Schneider M., Müller C.E., Vázquez S., Griñán-Ferré C., Pallàs M. (2022). NMDA receptor antagonists reduce amyloid-β deposition by modulating calpain-1 signaling and autophagy, rescuing cognitive impairment in 5XFAD mice. Cell. Mol. Life Sci..

[209] Ebrahimi Z., Talaei S., Aghamiri S., Goradel N.H., Jafarpour A., Negahdari B. (2020). Overcoming the blood–brain barrier in neurodegenerative disorders and brain tumours. IET Nanobiotechnology.

[210] Aragón-González A., Shaw P.J., Ferraiuolo L. (2022). Blood–Brain Barrier Disruption and Its Involvement in Neurodevelopmental and Neurodegenerative Disorders. Int. J. Mol. Sci..

[211] Song Q., Li J., Li T., Li H. (2024). Nanomaterials that Aid in the Diagnosis and Treatment of Alzheimer’s Disease, Resolving Blood–Brain Barrier Crossing Ability. Adv. Sci..

[212] Xie J., Shen Z., Anraku Y., Kataoka K., Chen X. (2019). Nanomaterial-based blood-brain-barrier (BBB) crossing strategies. Biomaterials.

[213] Wu D.-D., Salah Y.A., Ngowi E.E., Zhang Y.-X., Khattak S., Khan N.H., Li T., Guo Z.-H., Wang Y.-M., Ji X.-Y. (2023). Nanotechnology prospects in brain therapeutics concerning gene-targeting and nose-to-brain administration. iScience.

[214] Nunes D., Loureiro J.A., Pereira M.C. (2022). Drug Delivery Systems as a Strategy to Improve the Efficacy of FDA-Approved Alzheimer’s Drugs. Pharmaceutics.

[215] Peng Y., Jin H., Xue Y.-H., Chen Q., Yao S.-Y., Du M.-Q., Liu S. (2023). Current and future therapeutic strategies for Alzheimer’s disease: An overview of drug development bottlenecks. Front. Aging Neurosci..

